# Integrated single-molecule real-time sequencing and RNA sequencing reveal the molecular mechanisms of salt tolerance in a novel synthesized polyploid genetic bridge between maize and its wild relatives

**DOI:** 10.1186/s12864-023-09148-0

**Published:** 2023-01-31

**Authors:** Xiaofeng Li, Xingyu Wang, Qiangqiang Ma, Yunfeng Zhong, Yibo Zhang, Ping Zhang, Yingzheng Li, Ruyu He, Yang Zhou, Yang Li, Mingjun Cheng, Xu Yan, Yan Li, Jianmei He, Muhammad Zafar Iqbal, Tingzhao Rong, Qilin Tang

**Affiliations:** 1 Sichuan Agricultural University, Chengdu, 611130 China; 2 Chengdu Research Base of Giant Panda Breeding, Chengdu, 61130 China; 3 Mianyang Teachers’ College School of Urban and Rural Construction and Planning, Mianyany, 621000 China; 4 Institute of Qinghai-Tibetan Plateau, Southwest Minzu University, Chengdu, 610041 China; 5 Sericulture Research Institute, Sichuan Academy of Agricultural Sciences, Nanchong, 637000 China; 6 Crop Research Institute, Sichuan Academy of Agricultural Sciences, Chengdu, 611041 China

**Keywords:** SMRT-sequencing, RNA-sequencing, *Zea mays*, *Tripsacum dactyloides*, *Zea perennis*, Genetic bridge, Salt tolerance

## Abstract

**Background:**

*Tripsacum dactyloides* (2*n* = 4*x* = 72) and *Zea perennis* (2*n* = 4x = 40) are tertiary gene pools of *Zea mays* L. and exhibit many abiotic adaptations absent in modern maize, especially salt tolerance. A previously reported allopolyploid (hereafter referred to as MTP, 2*n* = 74) synthesized using *Zea mays*, *Tripsacum dactyloides,* and *Zea perennis* has even stronger salt tolerance than *Z. perennis* and *T. dactyloides*. This allopolyploid will be a powerful genetic bridge for the genetic improvement of maize. However, the molecular mechanisms underlying its salt tolerance, as well as the key genes involved in regulating its salt tolerance, remain unclear.

**Results:**

Single-molecule real-time sequencing and RNA sequencing were used to identify the genes involved in salt tolerance and reveal the underlying molecular mechanisms. Based on the SMRT-seq results, we obtained 227,375 reference unigenes with an average length of 2300 bp; most of the unigenes were annotated to *Z. mays* sequences (76.5%) in the NR database. Moreover, a total of 484 and 1053 differentially expressed genes (DEGs) were identified in the leaves and roots, respectively. Functional enrichment analysis of DEGs revealed that multiple pathways responded to salt stress, including “Flavonoid biosynthesis,” “Oxidoreductase activity,” and “Plant hormone signal transduction” in the leaves and roots, and “Iron ion binding,” “Acetyl-CoA carboxylase activity,” and “Serine-type carboxypeptidase activity” in the roots. Transcription factors, such as those in the WRKY, B3-ARF, and bHLH families, and cytokinin negatively regulators negatively regulated the salt stress response. According to the results of the short time series-expression miner analysis, proteins involved in “Spliceosome” and “MAPK signal pathway” dynamically responded to salt stress as salinity changed. Protein–protein interaction analysis revealed that heat shock proteins play a role in the large interaction network regulating salt tolerance.

**Conclusions:**

Our results reveal the molecular mechanism underlying the regulation of MTP in the response to salt stress and abundant salt-tolerance-related unigenes. These findings will aid the retrieval of lost alleles in modern maize and provide a new approach for using *T. dactyloides* and *Z. perennis* to improve maize.

**Supplementary Information:**

The online version contains supplementary material available at 10.1186/s12864-023-09148-0.

## Background

Climate change and environmental change pose a significant threat to crop production worldwide. Soil salinization is widespread, and climate change and irrigation-dependent cultivation practices have contributed to increases in soil salinization. Salinity can induce imbalances in intracellular osmotic regulation and result in plant dehydration. In addition, excessive salt absorption can affect the biological functions of plants—such as accelerating the degradation of chlorophyll, inhibiting photosynthesis, decreasing the growth rate—and inhibit the ability to uptake nutrients and other various metabolic reactions [1, 2]. Maize (*Zea mays* L.) provides more than 50% of the world’s calories annually [3].

Domestication has resulted in a significant loss of genetic diversity in cultivated maize, and this has decreased the resistance of maize plants to abiotic and biotic stress [4, 5]. Maize is a glycophytic crop that is highly sensitive to salinity; thus, soil salinization has led to a gradual decrease in the area of arable land as well as maize production [6]. To improve the salt tolerance of maize, salt-tolerant genes that regulate osmotic regulation, sodium ion homeostasis, potassium ion homeostasis, and chloride homeostasis have been cloned from existing maize germplasm [3, 7, 8]. Nevertheless, existing germplasm resources are currently unable to meet the demands of breeding. The wild relatives of maize, such as teosinte and *T. dactyloides*, retain high levels of genetic diversity and are well adapted to harsh environments [9, 10]. Therefore, identifying and utilizing salt-tolerance genes in wild relatives is essential for the breeding of salt-tolerant maize varieties.

All species except for maize in the genus *Zea* are referred to as teosinte (2*n* = 2*x* = 20 or 2*n* = 4*x* = 40); given that only diploid species (2*n* = 2*x* = 20) are cross-compatible with maize, they have been used to expand maize germplasm resources [11]. *Z. perennis*, the only tetraploid species (2*n* = 4*x* = 40) in the genus *Zea*, is immune to a variety of maize viruses [12, 13]. *T. dactyloides* is a tertiary gene pool for maize, and it shows high resistance to biotic [14–17] and abiotic stress [18, 19]. *T. dactyloides* is also considered highly tolerant of drought and salt stress (https://www.fnps.org/). Some studies have confirmed that hybrids (*Z. mays* × *T. dactyloides*) show high salt tolerance compared with maize lines [20, 21]. Overall, *T. dactyloides* and teosinte are thought to be important genetic reservoirs of diversity for the genetic improvement of modern maize [22, 23]. However, high ploidy levels and reproductive isolation severely restrict gene flow between maize and these two wild relatives under natural circumstances [24, 25].

Recreating polyploids between crops and wild relatives as an intermediate bridge may facilitate access to novel traits; this is also an effective approach for overcoming distant hybridization barriers and introducing foreign genes into crops from wild relatives [26, 27]. Three types of genetic bridges between *T. dactyloides* and maize have been previously reported (i.e., 2*n* = 2*x* = 10Zm +18Td, 2*n* = 3x = 10Zm + 36Td, 2*n* = 4*x* = 20Zm +36Td) [25, 28, 29]. Although severe meiotic abnormalities and sterility were documented in the hybrid progeny, these genetic bridges still provide a possible pathway of gene flow from *Z. perennis* and *T. dactyloides* to maize. In recent years, the development of the genetic bridges of *Z. perennis* and *T. dactyloides* in maize breeding has been slow, and the lack of genomic and transcriptomic information increases the difficulty of conducting further research.

A new tri-species hybrid known as MTP (2*n* = 20 M+ 34 T + 20P = 74) was first described in 2019, and it contains the genomes of three different species (M, T, and P, which stand for autotetraploid maize, *T. dactyloides,* and *Z. perennis*, respectively) [30]. MTP, which is male-sterile, partly female-fertile, perennial, and vegetatively propagated (via aerial stems or mother stands), is free of the drawbacks of the aforementioned genetic bridges; as a result, we can obtain enough individuals of MTP to cross with maize and carry out experiments to evaluate stress resistance. DNA sequences have been eliminated from the genome of MTP during allohexaploidization, yet the genome of MTP is 2.96 times larger than that of maize [31, 32]. Moreover, we developed a series of introgression lines with high stress resistance and agronomic characteristics using MTP as the donor and maize as the recipient (unpublished data). Using MTP as a genetic bridge has facilitated the introduction of beneficial genes of *Z. perennis* and *T. dactyloides* into maize. However, due to the lack of genomic information for MTP, in-depth molecular biology research has not yet been conducted, and this severely restricts its potential to be used for the genetic improvement of maize.

Single-molecule real-time sequencing (SMRT-seq) provides an alternative approach for directly generating full-length transcripts, and it is suitable for species lacking genomic information [33]. This technology has been used to reveal the complexity of transcriptomes; it also has been combined with RNA-seq to reveal the mechanism of resistance to abiotic and biotic stress in non-model organisms [2, 34–36]. In this study, we used SMRT-seq and RNA-seq to analyze the transcriptome of MTP. Specifically, we aimed to (i) generate reference transcripts and clarify the complex composition of the transcriptome of MTP, (ii) identify differentially expressed genes (DEGs) involved in the response to salt stress, and (iii) characterize the key molecular pathways involved in the response to salt stress in the roots and leaves. Our study provides novel genetic resources for the genetic improvement of maize, elucidates the salt tolerance mechanism of MTP, and sheds new light on the utilization of *T. dactyloides* and *Z. perennis* for the genetic improvement of maize.

## Materials and methods

### Plant materials, growth conditions, and salt tolerance evaluation

The salt tolerance of allohexaploid MTP was evaluated; maize (inbred Mo17), *T. dactyloides*, and *Z. perennis* were used as controls. MTP was synthesized from intergeneric crosses of tetraploid *Z. mays* (2*n* = 4*x* = 40), tetraploid *T. dactyloides* (2*n* = 4*x* = 72), and tetraploid *Z. perennis* (2*n* = 4*x* = 40) [30]. *T. dactyloides* and maize inbred Mo17 were provided by the United States Department of Agriculture (USDA), and *Z. perennis* was obtained from the International Maize and Wheat Improvement Center (CIMMYT). Except for inbred Mo17, which was propagated via seeds, the other three materials were propagated via rootstock, and the rootstock was acquired from the experimental field of Sichuan Agriculture University (Chengdu, China) (30°26′N–31°26′N, 102°54′E–104°53′E).

Seedlings of maize, *T. dactyloides*, *Z. perennis*, and MTP at the same growth stage were cultivated in pots (21 cm in diameter and 15 cm in height) filled with vermiculite in a greenhouse (14 h of light at 28 °C and 10 h of darkness at 23 °C, 75% humidity); 1/2 Hoagland’s nutrient solution was applied to the pots to ensure that plants were in a normal physiological state before treatment. After pre-culture for 5 days, the plants were divided into two groups (control and salt stress); the control (CK) and salt stress (T) groups were irrigated with 1/2 Hoagland’s nutrient solution and 1/2 Hoagland’s nutrient solution containing 300 mM NaCl for 25 days, respectively. To ensure the consistency of the salt concentration, the solution was replenished every 3 days, and an excess of the solution was applied each time. Agronomic and physiological traits were determined every 5 days, and leaves and roots were individually harvested after the final treatment for ion content determination. There were three biological replicates per treatment.

### Physiological and biochemical indexes

Agronomic and physiological parameters were measured at 0, 5, 10, 15, 20, and 25 d after treatment, including the leaf chlorophyll content, relative water content (RWC), relative electric conductivity (REC), plant height (PH), and the content of ions (Na^+^ and K^+^). Leaves were sampled and weighed quickly to record the fresh weight. The samples were hydrated to full turgidity with deionized water for 4 h after removing the surface moisture and immediately weighed to obtain the turgid weight. The samples were dried in a hot air oven at 80 °C for 2 days for dry weight measurements [37]. The RWC was determined using the following equation:

$$\textrm{RWC}=\frac{\textrm{fresh}\ \textrm{weight}-\textrm{dry}\ \textrm{weight}}{\textrm{turgid}\ \textrm{weight}-\textrm{dry}\ \textrm{weight}}$$

Leaf samples (0.2 g) were collected from each treatment and rinsed three times with deionized water; each sample was then placed into cuvettes with a cap, and deionized water (30 mL) was added. The initial electrical conductivity (EC1) of the mixture was measured after incubation at 25 °C for 6 h. After boiling at 100 °C for 30 min, the mixture was cooled to room temperature to determine the final electric conductivity (EC2). REC was determined using the following equation:

$$\textrm{REC}=\frac{\textrm{EC}2-\textrm{EC}1}{\textrm{EC}2}$$

The leaf chlorophyll content was measured in SPAD units. Plant height (PH) was measured from the leaf tip to the base of the stem. Leaf and root samples were rinsed with deionized water and dried at 80 °C for 2 days to a constant weight on the final day of the treatments; dried leaf and root samples (100 mg) were ground to powder, and extractions were conducted with HNO_3_ for 90 min at 95 °C, followed by filtering with Whatman filter paper. The content of Na^+^ and K^+^ in the solution was determined using a Varian Spectra AA-10 atomic absorption spectrophotometer [38]. The relative values were determined using the following equation:

$$\textrm{Relative}\ \textrm{value}\ \textrm{of}\ \textrm{indexes}=\frac{\textrm{indexes}\left(\textrm{T}\right)}{\textrm{indexes}\left(\textrm{CK}\right)}$$

The indexes were obtained for the K^+^ content, Na^+^ content, and Na^+^: K^+^ ratio. The data were analyzed by ANOVA in Excel 2019 (Microsoft, Redmond, USA) and SPSS 27.0 (SPSS, Chicago USA)software and plotted using OriginLab 2022 (Originlab, MA, USA) and R4.2.1.

### SMRT-seq library construction and sequencing

To obtain more comprehensive full-length transcriptome sequence information, leaf, stem, root, ear, and tassel tissues of MTP with the same weight at multiple growth stages were sampled for iso-sequencing. Samples rinsed with cold deionized water were quick-frozen in liquid nitrogen, and total RNA was extracted from each tissue using an RNAeasy Plant Mini Kit following the manufacturer’s instructions, followed by treatment with DNase I (Thermo Scientific, USA). The quantity and integrity of total RNA were assessed using a NanoDrop 2000 spectrophotometer and Agilent 2100 Bioanalyzer (Agilent, USA). Equal quantities of total RNA from each sample were pooled for library construction. Three size-fractionated libraries (1–2 kB, 2–3 kB, and 3–6 kB) were constructed for sequencing. Iso-sequencing was performed using the SMRT cells of Pacific Bio-systems to generate a high-quality, full-length reference transcriptome via Novogene Bioinformatics Technology Co., Ltd. (Beijing, China). Data were analyzed by running the Iso-Seq pipeline in the SMRT-Analysis software package.

Subreads generated by PacBio sequencing were clustered into circular consensus sequences (CCS) and non-CCSs by the Isoseq3 cluster program. Isoseq3 polish was used to obtain high-quality (HQ) isoforms and low-quality (LQ) isoforms with ≥99% accuracy. Interactive clustering of error correction (ICE) was applied to all full-length (FL) transcripts to generate the consensus transcripts by approaching clustering. Finally, CD-HIT-EST was used to remove redundancies to produce non-redundant high-quality transcripts. The transcripts were used in subsequent analyses.

Gene functions were annotated based on the following public databases: NCBI non-redundant nucleotide sequences (Nt), NCBI non-redundant protein sequences (Nr), Protein family (Pfam), Clusters of Orthologous Groups of proteins (KOG/COG), Swiss-Prot, Kyoto Encyclopedia of Genes and Genomes (KEGG), and Gene Ontology (GO) databases (E-value <1e-5) [39–41].

### Illumina RNA-seq library construction and sequencing

Except for the concentration of salt, the growth conditions were consistent with those used in evaluating the salt tolerance of MTP. All plants were divided into five groups with three replicates per group and treated with 1/2 Hoagland’s nutrient solution containing 0, 100, 200, 300, and 400 mM NaCl for 72 h. Leaves and roots were sampled at 72 h; there were three biological replicates for each treatment. All the above samples were quickly washed in 70% ethanol, frozen immediately in liquid nitrogen, and stored at − 80 °C in an ultra-low temperature freezer. The samples were denoted as “Tissue-Concentration”; for example, L-100 indicates leaves that were subjected to 100 mM NaCl stress. Total RNA was extracted using an RNeasy Plant Mini Kit per the manufacturer’s protocol, followed by treatment with DNase I (Thermo Scientific, USA), which resulted in the production of 30 RNA libraries. Libraries were sequenced using the Illumina Hiseq2500 platform to generate 150-bp paired-end reads. Library preparation and sequencing services were also performed by Novogene Bioinformatics Technology Co., Ltd. (Beijing, China).

To investigate the gene expression profiles of MTP under saline stress, Illumina clean reads were mapped to the full-length transcripts using Bowtie2. FPKM values were then used to normalize the reads from RNA-seq to identify differentially expressed genes (DEGs) between the groups being compared using RESM 1.2.15 and DESeq2 software. The *P*-value was assigned to each gene and adjusted using the Benjamini and Hochberg approach to control the false discovery rate. Genes with *q* ≤ 0.05 and | log_2_ ratio | ≥ 1 were DEGs, and gene expression pattern analysis was conducted using Short Time-series Expression Miner (STEM) software [42]. TBtools software v1.106 (China) was used to visualize the results of the KEGG, GO, and DEG analyses [43].

### Alternative splicing analysis

rMATS (3.2.5) software with default parameters was used to perform alternative splicing (AS) analysis of non-redundant sequences. AS events in MTP were compared with those in maize and *T. dactyloides* [19].

### Validation of RNA-seq data by quantitative RT-PCR

We used qRT-PCR to validate the reliability of the relative expression levels estimated via RNA-seq. qRT-PCR was performed using the “SYBR” Premix Ex TaqTM kit (Takara, Japan) on a Roche LightCycler480 instrument. Glyceraldehyde 3-phosphate dehydrogenase (*GAPDH*) was used as an internal control. Primers were designed using an online program from NCBI. The relative abundance of transcripts was determined using the 2^-ΔΔCT^ method [44]. Experiments were conducted three times to ensure accuracy.

### Protein–protein interaction (PPI) network construction

Orthologous pairs between *Z. mays* and MTP were obtained using Blastx (E-value <1e-10) against the STRING database (http://string-db.org/). The PPI network was constructed for the DEGs of MTP using *Z. mays* as a reference species; the network was visualized using Cytoscape 3.8.2 (https://cytoscape.org/).

## Results

### Phenotypic evaluation of the salt tolerance of MTP

**Fig. 1:**
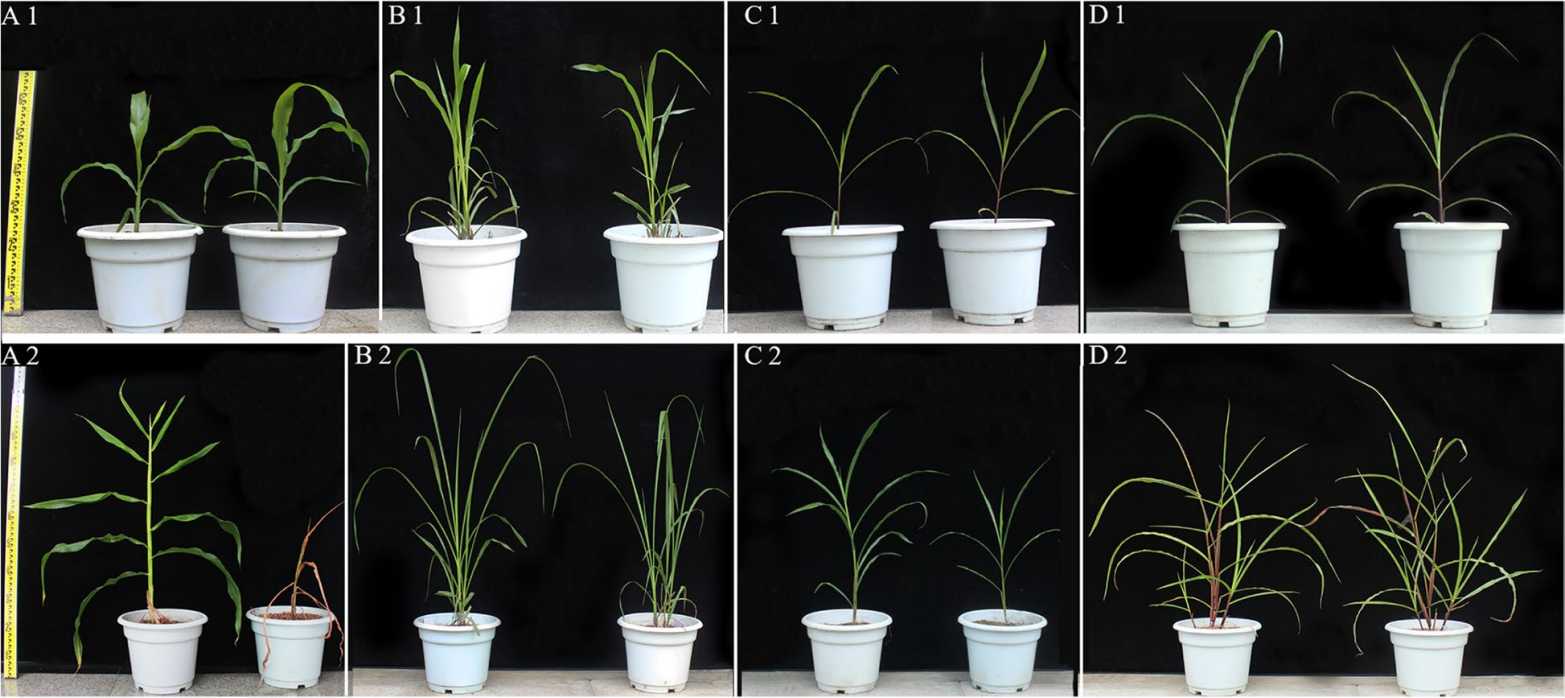
Morphological characteristics of plants under normal conditions and salt stress. **A1**–**D1** show *Z. mays* (Mo17), *T. dactyloides*, *Z. perennis*, and MTP under normal conditions, respectively. **A2**–**D2** show *Z. mays* (Mo17), *T. dactyloides*, *Z. perennis*, and MTP under normal conditions (left) and salt stress (right) after 25 days, respectively. Normal and salt stress treatments involved treatment with 1/2 Hoagland’s nutrient solution and 1/2 Hoagland’s nutrient solution containing 300 mM NaCl, respectively

**Fig. 2:**
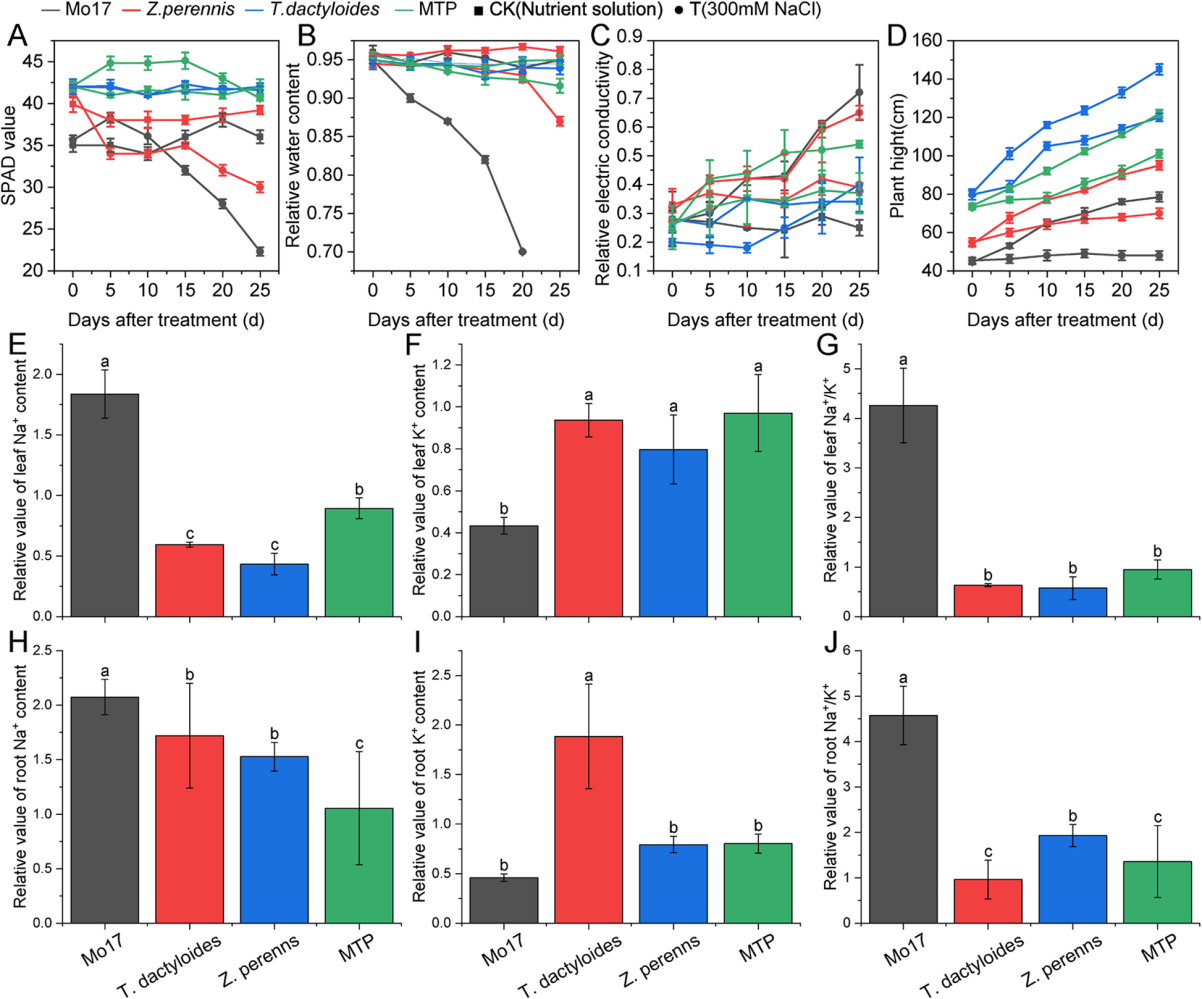
Growth and physiological indices. **A**–**D** indicate changes in leaf SPAD (chlorophyll content), relative water content, relative electric conductivity, and plant height under salt stress, respectively. **E**–**G** indicate the relative value (T/CK) of the leaf K^+^ content, Na^+^ content, and Na^+^: K^+^ ratio, respectively. **H**–**J** indicate the relative value of the root K^+^ content, Na^+^ content, and Na^+^: K^+^ ratio, respectively. Black, red, blue, and green indicate the three parents Mo17, *T. dactyloides*, *Z. perennis*, and tri-species hybrid MTP, respectively

Salinization occurs when the salt concentration is higher than 50 mM, as such salt concentrations are known to severely inhibit the growth of maize. In this study, 300 mM NaCl was used to evaluate the salt tolerance of MTP, and the parents of MTP, such as Mo17 (used as a replacement for tetraploid *Z. mays*), *Z. perennis*, and *T. dactyloides*, were used as controls (see Materials and methods). All plants grew well under normal conditions (Fig. 1A1–D1) but were affected to varying degrees under salt stress (Fig. 1A2–D2). Under salt stress, Mo17 experienced extensive changes (Fig. 1A2): the content of chlorophyll (SPAD) and the leaf water content decreased sharply, the relative electric conductivity increased sharply, growth in the height of maize ceased, and plants even died after 20 days of exposure to salt stress (Fig. 2A–D). The growth rate of *Z. perennis* and *T. dactyloides* slightly slowed under salt stress, and signs of salt injury were less pronounced in these two wild relatives compared with Mo17. There were no differences in the morphology of MTP between normal conditions and salt stress (Figs. 1 and 2A–D); even the number of tillers of MTP increased under salt stress (Fig. 1D2).

The maintenance of a low Na^+^ content and Na^+^: K^+^ ratio is known to be essential for salt tolerance in plants [45]. We therefore characterized Na^+^ and K^+^ homeostasis in the leaves and roots of Mo17, *T. dactyloides*, *Z. perennis*, and MTP. We found that the K^+^ content of Mo17 in the leaves was significantly lower than that in other plants, and the Na^+^ content and Na^+^:K^+^ ratio were significantly higher in both the leaves and roots of Mo17 than in the leaves and roots of the other three plants. The Na^+^ content, and Na^+^: K^+^ ratio in the leaves and roots were significantly lower in MTP, *T. dactyloides*, and *Z. perennis* than in maize (Fig. 2E–J). 

### Full-length transcript construction and statistical and functional annotation analysis

**Fig. 3:**
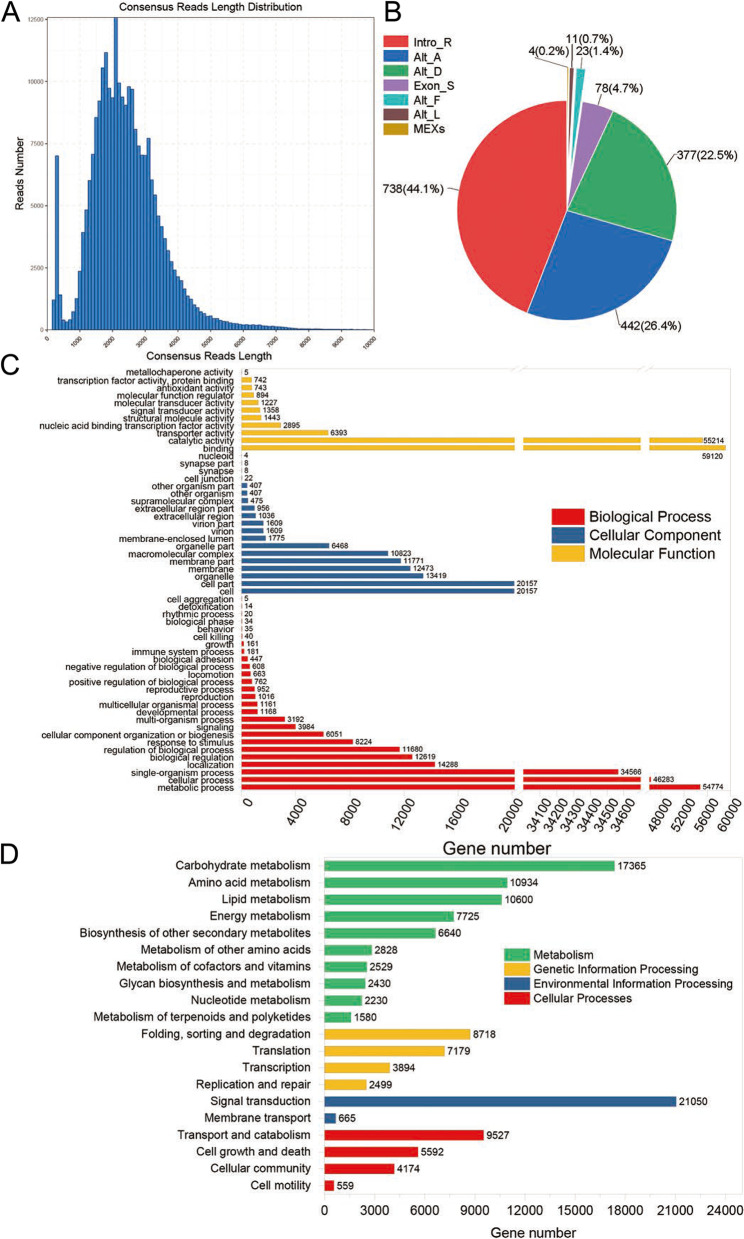
SMRT sequencing data statistics. **A** The length distribution of consensus reads. **B** AS types (Intro-R: Intron Retention, Alt-A: Alternative Acceptor, Alt-D: Alternative Donor, Exon-S: Exons skipping, Alt-F: Alternative First Exons, Alt-L: Alternative Last Exons, MEX: Mutually Exclusive Exons) in MTP. **C** GO functional classification. **D** KEGG pathway classification

Using Iso-Seq SMRT Pipe v2.3.0, we obtained a total of 244,418 polished consensus sequences, which were mainly distributed from 1000 bp to 4000 bp, with a base call accuracy of ≥99% (Fig. 3A). A total of 227,375 full-length non-redundant unigenes were generated by CD-HIT software (Table S1). Seven patterns of AS comprising 1673 AS events were detected in MTP; intron retention (Intro-R) was the most common, followed by alternative acceptor (Alt-A), alternative donor (Alt-D), exon skipping (Exon-S), alternative first exons (Alt-F), alternative last exons (Alt-L), and mutually exclusive exons (MEXs) (Fig. 3B). These findings are consistent with the results of previous studyof AS in *T. dactyloides* and maize that employed Iso-Seq technology [19].

A total of 94.73% (215,379/227,375) of the unigenes were annotated against four protein databases (NR, SwissProt, Nt, and Pfam) using Blast (E-value ≤1e-10). Sequence homology alignment against the NR database revealed that most of the unigenes had significant hits in *Z. mays* (76.49%), followed by *Sorghum bicolor* (6.01%,), *Setaria* (1.80%), and *Oryza sativa* (0.95%). Unigenes were mainly annotated to 56 GO terms (Fig. 3C). KEGG enrichment analysis showed that most of these unigenes were enriched in the signal transduction pathway, followed by the carbohydrate metabolism pathway (Fig. 3D).

Transcription factors (TFs) and transcriptional regulators (TRs) play critical roles in plant development. In this study, a total of 8317 TFs belonging to 48 families and 2658 TRs belonging to 21 families were predicted using iTAK software. CH3, MYB, and HB were the most common in all TF families, and SNF2, AUX/IAA, and SET were the most common in all families of TRs (Fig. S1). A total of 12,982 lncRNAs with an average length 788 bp (ranging from 0.2 kb to 1.5 kb) were identified (Fig. S2), and 1826 lncRNAs showed high sequence similarity with lncRNAs in maize (identity > 80%, E-value = 1e-5) (Fig. S4H).

### Comparative transcriptome analysis between MTP and other species

Unigenes of MTP were mapped to the maize reference genome (RefGen-v4) using GMAP software (version 2018-07-04) with the parameters -min-trimmed-coverage 85% and -min-identity 85%; other parameters were set to their default values. We found that 83.76% of unigenes could be mapped to the maize reference genome; this was substantially lower than the mapping ratio (98.04%) between maize and *T. dactyloides* [19]. A total of 132,912 unigenes of MTP were mapped to 18,082 maize gene models (Fig. S4A, E); 3668 unigenes of MTP were uniquely mapped to maize genes, and 129,244 unigenes were multiply mapped to maize genes. On average, 7.35 unigenes were mapped to a single maize gene model, and up to 1277 unigenes were mapped to a single maize gene model (Zm00001d017274), which encoded a phenylalanine ammonia-lyase. Fig. S3B, C, D, and E show the densities of maize genes aligned to *T. dactyloides*, teosinte (*Zea nicarguensis*), *Sorghum*, and MTP genes across all maize chromosomes. Maize–sorghum and maize–sorghum–MTP syntenic gene pairs ensured the confidence of these mappings (Fig. S3F, G). Figure S3H shows the densities of the lncRNA in MTP. Unmapped unigenes were aligned in the NCBI RefSeq using BlastX (Identity ≥70%, E-value ≤1e-5) to predict homologous species. Only a few genes were aligned to those in other *Zea* species, such as *Zea mexicana*, *Zea diploperennis*, and *Zea parviglumis*, and two sequences that encode phytoene synthase and chitinase were aligned to *T. dactyloides* (Tables S2 and S3). Fewer genes were aligned to *Z. perennis* and *T. dactyloides*, which might stem from the lack of genetic information for *Z. perennis* and *T. dactyloides* in current databases.

**Fig. 4:**
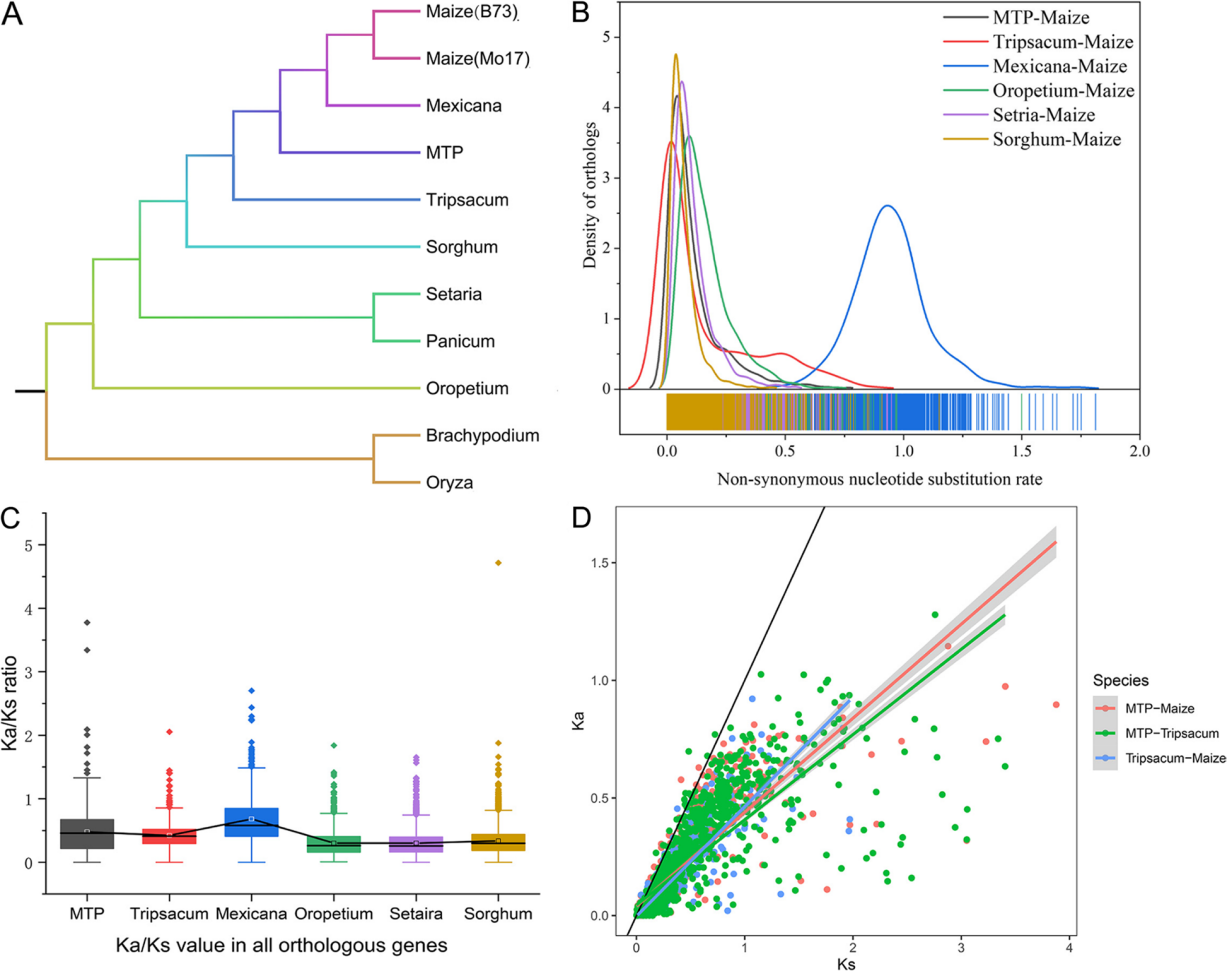
Synonymous substitution rates and non-synonymous substitution rates across nine-related grass species. **A** Phylogenetic relationships of the 11 species employed in this study. Red, green, and orange indicate target species, background species, and outgroups, respectively. **B** Distribution of synonymous substitution rates in orthologous gene groups across each target and background species. **C** Distribution of Ka/Ks ratios for orthologous genes conserved in MTP, *T. dactyloides*, *Z. mexicana*, *Oropetium*, *Setaria*, and *Sorghum*. **D** Comparative distribution of Ka/Ks ratios of orthologs between MTP and Maize, *Tripsacum* and maize, and MTP and *T. dactyloides*

A total of 6815 orthologous groups were identified in nine grass species according to maize (Mo17 and B73)–MTP–*T. dactyloides* ortholog relationships (Table S4), and the phylogenetic relationships of the nine grass species are shown in Fig. 4A. *Sorghum*, *Setaria*, *Panicum*, *Oropetium*, and *Brachypodium* were used as outgroups. The orthologous gene set consisted of 3122 one-to-one, 4430 one-to-many, 989 many-to-one, and 1396 and 1249 many-to-many orthologous gene sets between B73 and MTP due to whole-genome duplication. The orthologous gene set between Mo17 and MTP did not differ greatly from that between B73 and MTP (Table S5). Ka/Ks has been used to determine differences in the strength of selection on proteins. In this study, single-copy ortholog gene pairs were identified using maize as a target species and other species as background species, and these were used to calculate Ka and Ks values. The overall distributions of Ka values are presented in Fig. 4B. MTP genes tended to have a higher synonymous substitution ratio than average *T. dactyloides* genes (using maize as the background species) (Fig. 4C). Only a few genes had high Ka/Ks ratios (> 1) between maize and MTP (Fig. 4D). The number of orthologous gene pairs between MTP and maize with Ka/Ks ratios > 1 was 37, and the number of such pairs between MTP and *T. dactyloides* was 36. All the genes in MTP with high Ka/Ks values were involved in biotic stress-related processes.

### Analysis of DEGs under salt stress in MTP

A total of 119 million raw reads were generated using the Illumina high-throughput platform, and there were a total of 115 million high-quality clean reads after quality control (Table S6). Clean reads were mapped to the full-length transcripts of MTP via Bowtie2 software (mismatch = 0); an average of 78.92% of the clean reads were uniquely mapped to the full-length transcripts of MTP (Table S6). Fragments per kilobase per million mapped fragments (FPKM) were calculated to characterize gene expression patterns. The FPKM distribution of all samples is shown in Fig. S4. As the concentration of salt increased, the proportion of highly expressed genes in leaves increased; the proportion of highly expressed genes decreased in the roots, and this decrease was most noticeable at the high salt concentration (400 mM). The correlation heatmap in Fig. S5 indicates the high reproducibility among biological replications of each sample; there was a weak correlation between leaves and roots. The number of DEGs tended to increase as the salt concentration increased in both the leaves and roots. DEGs between leaves and roots were more numerous and showed tissue-specific expression patterns (Fig. S6).

**Fig. 5:**
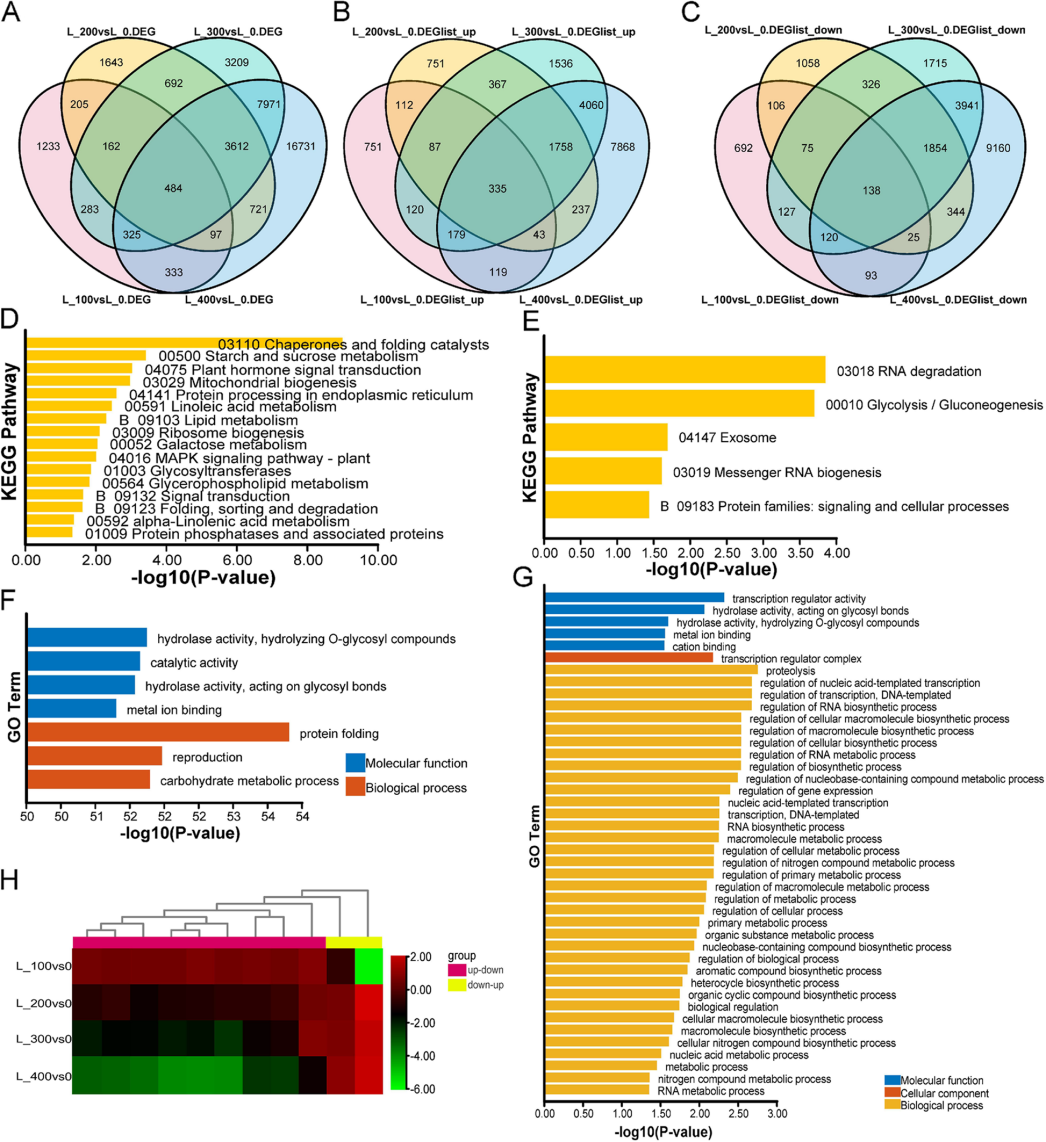
Functional enrichment of DEGs in leaves. **A** Venn diagram of co-induced DEGs under different concentrations of salt stress. **B** Genes with up-regulated expression. **C** Genes with down-regulated expression. **D** KEGG enrichment analysis of genes with up-regulated expression. **E** KEGG enrichment analysis with down-regulated expression. **F** GO enrichment analysis of genes with up-regulated expression. **G** GO enrichment analysis of genes with down-regulated expression. **H** Expression patterns of genes in (**A**) but not in (**B**) and (**C**). The deep pink group indicates genes with up-regulated expression under low salt concentrations and down-regulated expression under high salt concentrations; the yellow group indicates genes with down-regulated expression under low salt concentrations and up-regulated expression under high salt concentrations

**Fig. 6:**
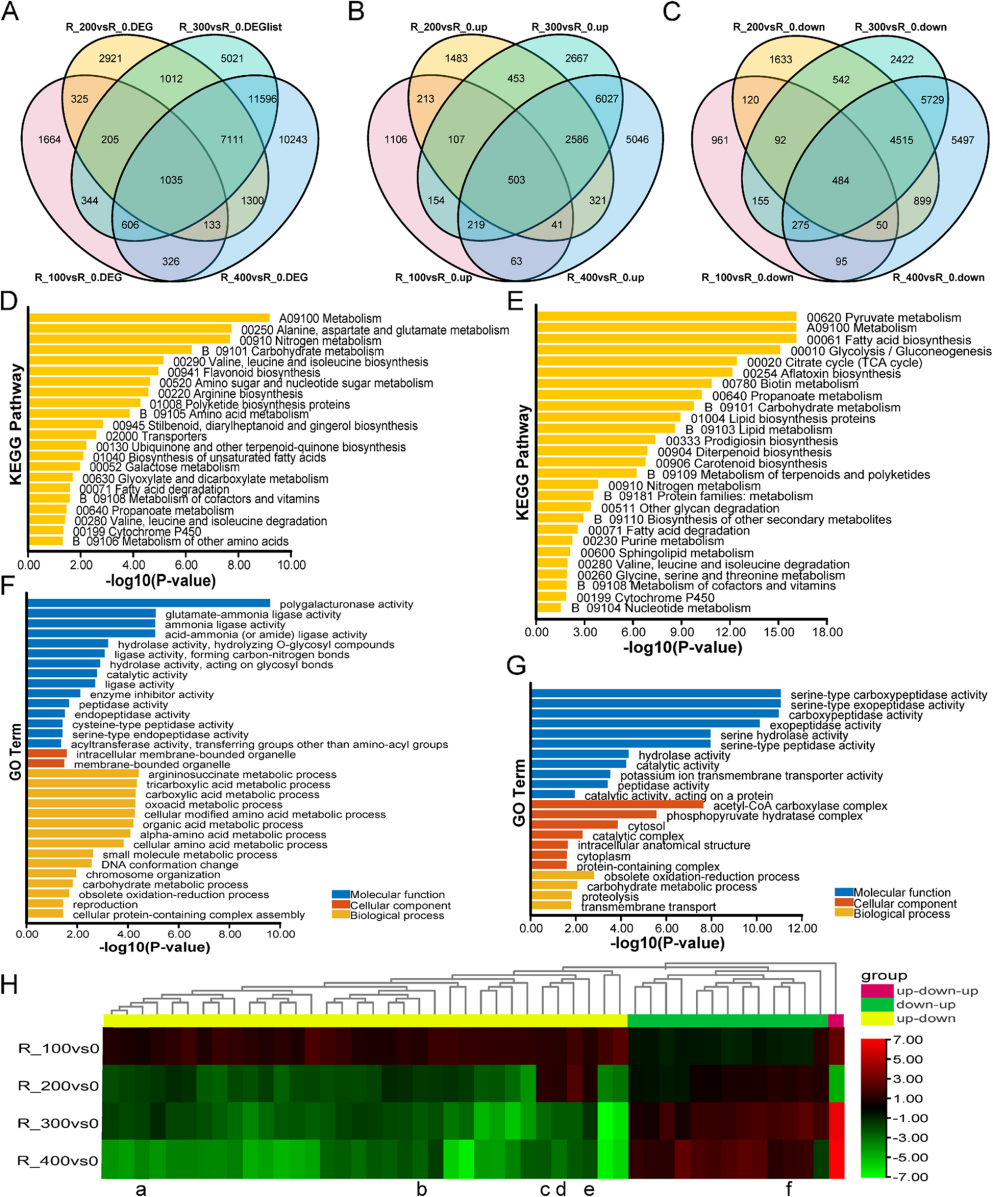
Functional enrichment of DEGs in roots. **A** Venn diagram of common DEGs under different salt concentrations. **B** Venn diagram of genes with up-regulated expression. **C** Venn diagram of genes with down-regulated expression. **D** KEGG enrichment analysis of genes with up-regulated expression. **E** KEGG enrichment analysis of genes with down-regulated expression. **F** GO enrichment analysis of genes with up-regulated expression. **G** GO enrichment analysis of genes with down-regulated expression. **H** Gene expression patterns of genes in (**A**) but not in (**B**) and (**C**). The deep pink group indicates genes with up-regulated expression under low salt concentrations and high salt concentrations but down-regulated expression under 200 mM NaCl. The green group indicates genes with down-regulated expression under low salt concentrations and up-regulated expression under high salt concentrations. The yellow group indicates genes with up-regulated expression under low salt concentrations and down-regulated expression under high salt concentrations. a, b, c, d, e, and f indicate WRKY, B3-ARF, ARR-A, ARR-A, bHLH, and LOB family TFs or response regulators, respectively

A total of 37,701 (leaves) and 43,842 DEGs (roots) were identified under salt stress compared with the control (Figs. 5A and 6A). The expression of 484 DEGs in leaves was co-induced under different salt concentrations, including 335 up-regulated genes and 138 down-regulated genes (Fig. 5B,C). KEGG enrichment analysis showed that up-regulated genes were enriched in 16 different metabolic pathways, including pathways involved in “Energy metabolism,” “Lipid metabolism,” “Protein behavior,” and “Plant signal transduction” (Fig. 5D). Down-regulated genes were enriched in pathways involved in “RNA degradation,” “Glycolysis/gluconeogenesis,” “Exosome,” “Messenger RNA biogenesis,” and “Signaling and cellular processes” (Fig. 5E). GO enrichment analysis results revealed that up-regulated genes were only enriched in seven GO terms (Fig. 5F); however, 138 down-regulated genes were enriched in 42 GO terms, and five of these GO terms were the same for both up-regulated genes and down-regulated genes, including “hydrolase activity acting on glycosyl bonds,” “hydrolase activity,” and “metal ion binding” (Fig. 5G). Direct acyclic graph (DAG) analysis revealed that several genes were involved in “Oxidoreductase activity,” “Glutathione gamma-glutamyl cysteinyl transferase activity,” and “Phosphatidylinositol 3-kinase binding” (Fig. S7).

The expression of 11 co-induced DEGs was not always up-regulated or down-regulated under different concentrations of salt stress (Fig. 5H). The expression of nine DEGs changed from being up-regulated to down-regulated as the salt concentration increased (Fig. 5H). Functional analysis revealed that these DEGs were mainly involved in “chlorophyll biosynthesis” and “Aquaporin PIP” and enriched in “Photosynthesis antenna proteins” and “Porphyrin and chlorophyll metabolism” pathways (Fig. S8). The other two DEGs exhibited the opposite pattern and were involved in “Elongation factor 2” and “Intron-binding protein Aquarius” (Fig. 5H).

A total of 1035 co-induced DEGs were identified under different salt concentrations in roots (Fig. 6A), including 503 up-regulated genes (Fig. 6B) and 484 down-regulated genes (Fig. 6C). A total of 503 up-regulated genes were enriched in 22 KEGG pathways (Fig. 6D) and 32 GO terms (Fig. 6F). A total of 484 down-regulated genes were enriched in 27 KEGG pathways (Fig. 6E) and 22 GO terms (Fig. 6G). Up-regulated and down-regulated genes were enriched in “Metabolism,” “Nitrogen metabolism,” “Carbohydrate metabolism,” “Valine, leucine and isoleucine biosynthesis,” “Fatty acid degradation,” “Metabolism of cofactors and vitamins,” and “Cytochrome P450.” In addition, up-regulated genes were especially enriched in “Polyketide synthases,” “Flavonoid biosynthesis,” and “Biosynthesis of unsaturated fatty acids” pathways. DAG analysis revealed that “Acetyl-CoA carboxylase activity,” “Oxidoreductase activity,” “Serine-type carboxypeptidase activity,” and “Iron ion binding” might play an important role in the response of roots to salt stress (Fig. S9).

The expression of 48 co-induced DEGs was not always up-regulated or down-regulated under different salt concentrations (Fig. 6H). Among these 48 unigenes, the yellow group comprised 34 genes with up-regulated expression under a low salt concentration but down-regulated expression under a high salt concentration, and these genes were enriched in the “Plant hormone signal transduction” pathway (Fig. S10). The green group comprised 13 genes that showed the opposite pattern, and these genes were mainly enriched in “Carbon fixation in photosynthetic organisms” and “Glycolysis/Gluconeogenesis” pathways (Fig. S11). Among these, four genes encoded ARF, bHLH, LOB, and WRKY family transcription factors (TFs), and two genes encoded ARR-A family cytokinin signal negative regulators (Fig. 6H).

### Changes in the expression profile of DEGs in leaves and roots under different salt concentrations

**Fig. 7:**
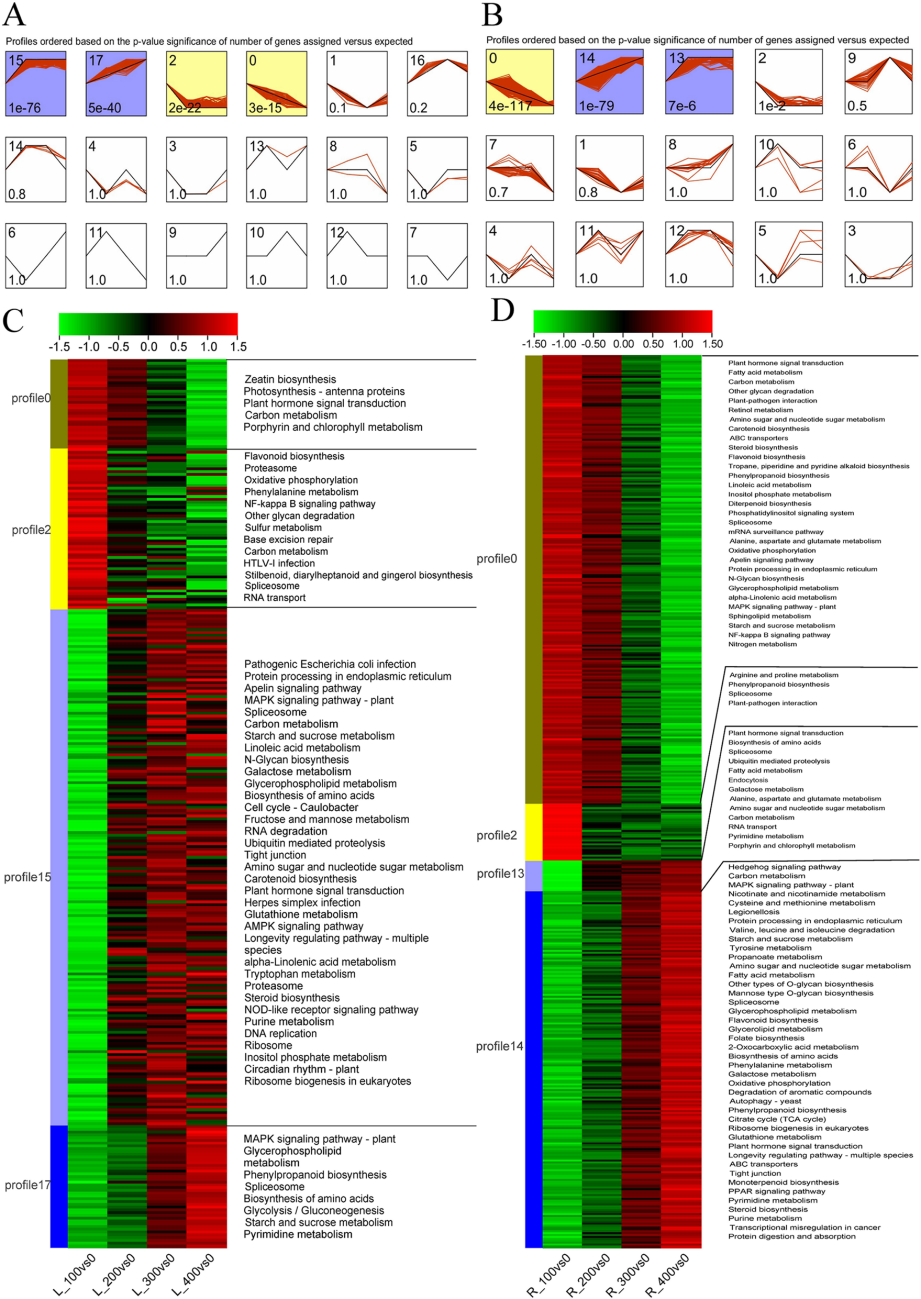
Changes in the expression patterns of DEGs in the leaves and roots according to STEM clustering (*P* ≤ 0.05). **A** Temporal expression profiles of DEGs in leaves. **B** Temporal expression profiles of DEGs in roots. **C** Heatmap of the expression levels of DEGs in the leaves and KEGG enrichment analysis. **D** Heatmap of the expression of DEGs in the roots and KEGG enrichment analysis. Profiles are ordered according to their *P*-values, and significantly different profiles are in different background colors (bottom left-hand corner numbers indicate *P*-values). The numbers in the upper left-hand corners indicate cluster or profile IDs, which are ordered according to their *P*-values

To characterize changes in the expression of DEGs under different salt concentrations, the expression of all DEGs in the leaves and roots was analyzed and visualized using STEM software. Four significant expression patterns of co-induced DEGs in leaves (Fig. 7A) (*P* ≤ 0.05) were identified by STEM analysis, and four similar expression patterns were detected in the roots (Fig. 7B). A heatmap of expression patterns and pathway enrichment are shown in Fig. 7C and D.

DEGs with expression Pattern 1 in leaves (i.e., profile 17 in Fig. 7A, expression level increased continuously as the salt concentration increased) were only enriched in eight KEGG pathways (Fig. 7C). DEGs with expression Pattern 1 in roots (i.e., profile 14 in Fig. 7B) were enriched in 42 KEGG pathways (Fig. 7D). Within these pathways, “MAPK signaling pathway,” “Spliceosome,” and “Starch and sucrose metabolism” were co-enriched in both the leaves and roots. The rest of the DEGs with expression pattern 1 in roots were also enriched in amino acid metabolism signaling pathways and the active oxygen scavenging system (Fig. 7D). DEGs with expression pattern 2 in leaves (i.e., profile 0 in Fig. 7A, expression level decreased continuously as the salt concentration decreased) were enriched in five pathways (Fig. 7C). DEGs with expression pattern 2 in roots (i.e., profile 0 in Fig. 7B) were mainly enriched in 31 pathways, and “Plant hormone signal transduction” and “Carbon metabolism” pathways were co-enriched in the leaves and roots (Fig. 7D). DEGs with expression pattern 3 (i.e., profile 15 in Fig. 7A, high expression level under high salt concentrations [200 mM, 300 mM, and 400 mM] but not under low salt concentrations) in leaves were enriched in 35 pathways (Fig. 7C). However, DEGs with expression pattern 3 (i.e., profile13 in Fig. 7B) in roots were only enriched in 13 pathways (Fig. 7D). “Plant hormone signal transduction,” “Biosynthesis of amino acids,” “Spliceosome,” “Ubiquitin mediated proteolysis,” and “Galactose metabolism” pathways were co-enriched in the leaves and roots. DEGs with expression pattern 4 in leaves (i.e., profile 2 in Fig. 7A, low expression level under high salt concentrations [200 mM, 300 mM, and 400 mM] but not under low salt concentrations) were enriched in 13 pathways (Fig. 7C). However, DEGs with expression pattern 4 in roots (profile 2 in Fig. 7B) were only enriched in four pathways (Fig. 7D). Among these pathways, only “Spliceosome” was co-enriched in both the leaves and roots.

A recent study showed that increases in the copy number of gene families involved in salt tolerance played a key role in the evolution of salt tolerance in *Achnatherum splendens* [46]. We identified paralogs of salt-tolerance genes in MTP. We also determined the copy numbers of salt-tolerant-related gene families in MTP and compared them with those in the perennial halophyte *Achnatherum splendens* Trin. We found that the copy numbers of the salt-tolerant-related gene families were 7.83 times higher in MTP than in *A. splendens* (Table S7). We then characterized the expression patterns of paralogous genes in MTP under salt stress; the expression of most of these genes was induced by salt stress, and significant differences in the expression of these genes were observed between the leaves and roots (Table S8). The high copy numbers of these gene families might contribute to the strong salt tolerance of MTP, and this might also be an important resource for the genetic improvement of maize.

### PPI networks revealed the complex potential regulatory pathways underlying the salt stress response in MTP

PPIs are critically important for the completion of complex molecular processes. We constructed a PPI network model for 484 DEGs in leaves and 1035 DEGs in roots using *Z. mays* as the reference species (Fig. S12). A total of seven subnetworks were obtained using DEGs in leaves (Fig. S8A–G). Most of the genes in the largest subnetwork (Fig. S12A) located at the core position encoded heat shock proteins (HSPs), including HSPA, HSP90, and HSPA4. Unigene-L26, which has the highest node degree, was annotated to the *T. dactyloides* genome sequence in the NT database and encodes the 40S ribosomal protein. This indicates that unigene-L26 might play an important role in this interaction network in response to salt stress. The other six subnetworks were involved in “Photosynthesis,” “Proteasome,” “Starch and sucrose metabolism,” “Metal ion binding,” “Oxidation-reduction,” and “MAPK signaling pathways” (Fig. S12B–G). The expression of all the genes in the subnetwork in Fig. S12B was down-regulated; functional annotation showed that all these genes were involved in “Photosynthesis,” indicating that the expression of genes involved in “Photosynthesis” was inhibited under salt stress.

A total of eleven subnetworks were obtained using the root DEGs (Fig. S12H–R). The largest subnetwork (Fig. S12H) was mainly involved in “Carbon metabolism,” “Biosynthesis of other secondary metabolism,” and “Starch and sucrose metabolism” pathways. The other subnetworks were involved in “Spliceosome,” “Arginine and proline metabolism,” “Sphingolipid metabolism,” “Steroid biosynthesis and terpenoid backbone biosynthesis,” “Amino sugar and nucleotide sugar metabolism,” “Galactose metabolism,” “Carotenoid biosynthesis,” “Fatty acid metabolism” (Fig. S12I–N), “Selenocompound metabolism,” “Aminoacyl-tRNA biosynthesis”, and “Protein processing in endoplasmic reticulum” (Fig. S12P–Q). The genes in subnetworks H (Fig. S12O) and K (Fig. S12R) were not annotated to any metabolic pathway, but they were mainly involved in ion transport, especially sodium and zinc ions in the Molecular Function Category of the GO database.

### Co-expressed genes in leaves and roots under salt stress

**Fig. 8:**
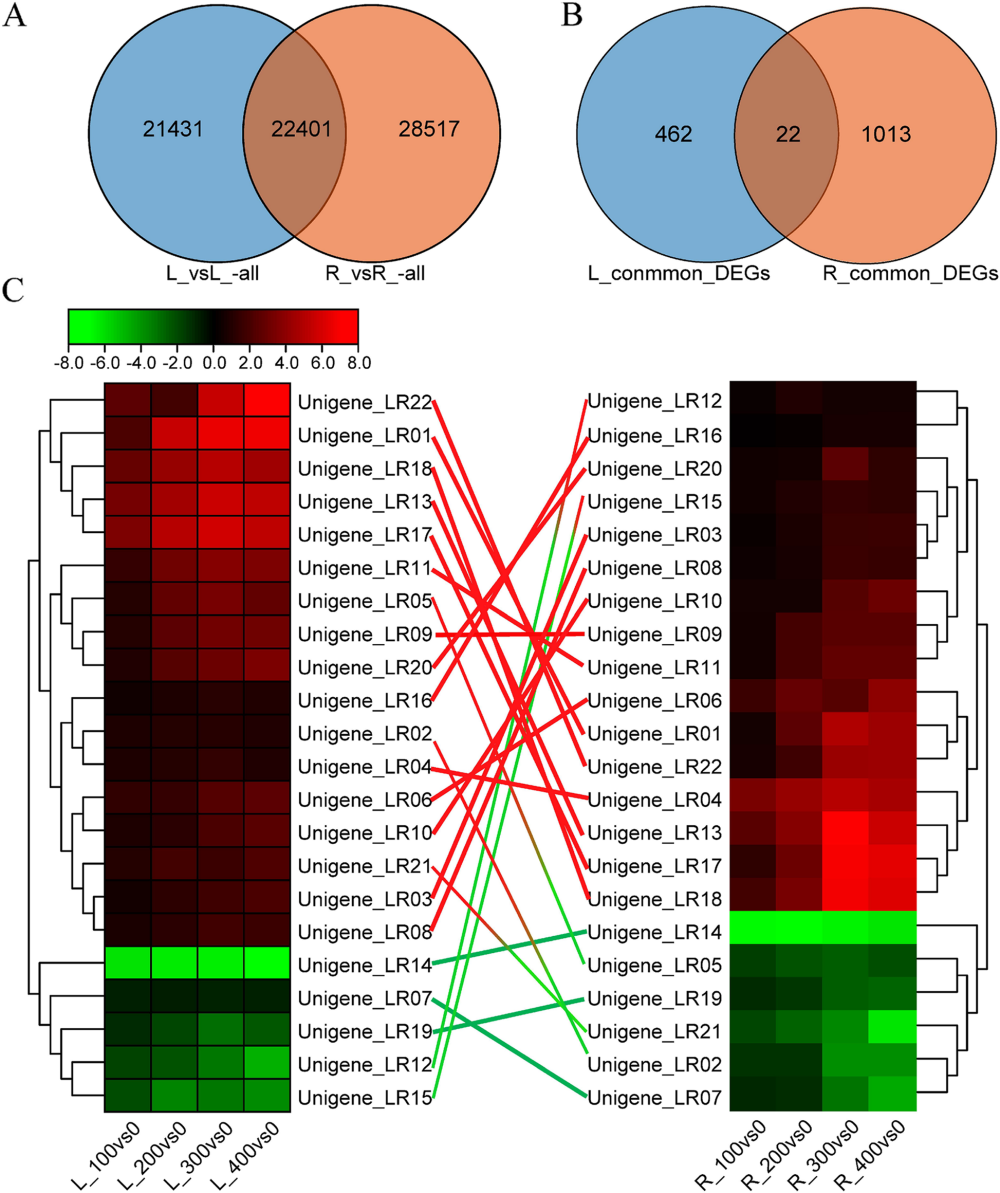
Co-expressed genes in the leaves and roots. **A** Venn diagram of all DEGs in the leaves and roots. **B** Venn diagram of DEGs under different salt concentrations. **C** Expression patterns of genes co-expressed in the leaves (left) and roots (right). The red connecting lines indicate genes showing up-regulated expression in both leaves and roots; green connecting lines indicate genes showing down-regulated expression in both leaves and roots; gradient lines (red-green) indicate genes showing up-regulated expression in leaves but down-regulated expression in roots; gradient lines (green-red) indicate genes showing down-regulated expression in leaves but up-regulated expression in roots

To determine whether genes were co-expressed simultaneously in both the leaves and roots under salt stress, the common DEGs between the leaves and roots were identified; a total of 22,401 DEGs were simultaneously expressed in the leaves and roots (Fig. 8A). However, we identified only 22 co-expressed genes in both the leaves and roots that were affected by variation in salt concentrations (Fig. 8B). The expression of 14 genes was up-regulated and the expression of three genes was down-regulated in both the leaves and roots under salt stress; the up-regulated genes were mainly involved in “Chitinase synthase” (Unigene-LR22), “Raffinose synthase” (Unigene-LR18, Unigene-LR13, Unigene-LR17), “3-deoxy-7-phosphoheptulonate synthase” (Unigene-LR06), “Splicing factor-arginine/serine-rich 1” (Unigene-LR03), and “Heat shock 70 kDa protein 1/8” (Unigene-LR08) metabolic pathways. The remaining genes were not annotated to any metabolic pathways in the KEGG database but were annotated to various pathways in the NR database, such as “P-loop containing nucleoside triphosphate hydrolase superfamily protein” (Unigene-LR01), “Phosphate regulatory homolog1” (Unigene-LR09), “Protein disulfide isomerase isoform X1” (Unigene-LR20), “*Zea mays* Cold shock protein 2” (Unigene-LR16), “Phosphate regulatory homolog1” (Unigene-LR11), and “Metabolite transport protein csbC” (Unigene-LR10). The three down-regulated genes were involved in “26S proteasome regulatory subunit N7 synthase pathway” (Unigene-LR14), “Ent-copalyl diphosphate synthase” (Unigene-LR19), and “Serine carboxypeptidase-like 51” (UnigeneLR07). The expression of Unigene-LR02, Unigene-LR05, and Unigene-LR21 was up-regulated in leaves but down-regulated in roots. and Unigene-LR02 was determined to encode an ARF TF; Unigene-LR05 was determined to encode “Abscisic stress-ripening protein 1.” Unigene-LR21 was involved in “Starch and sucrose metabolism pathway.” The expression of Unigene-LR12 and Unigene-LR15 was down-regulated in leaves but up-regulated in roots. Unigene-LR12 was involved in “Protein methylation/lysine catabolic process,” and Unigene-LR15 was annotated to the Uncharacterized homologous gene *LOC100304023* in *Z. mays* (Fig. 8C). These results suggest that the 17 genes showing the same expression patterns in leaves and roots play similar functional roles in the response to salt stress.

### Validation of gene expression patterns by qPCR

**Fig. 9:**
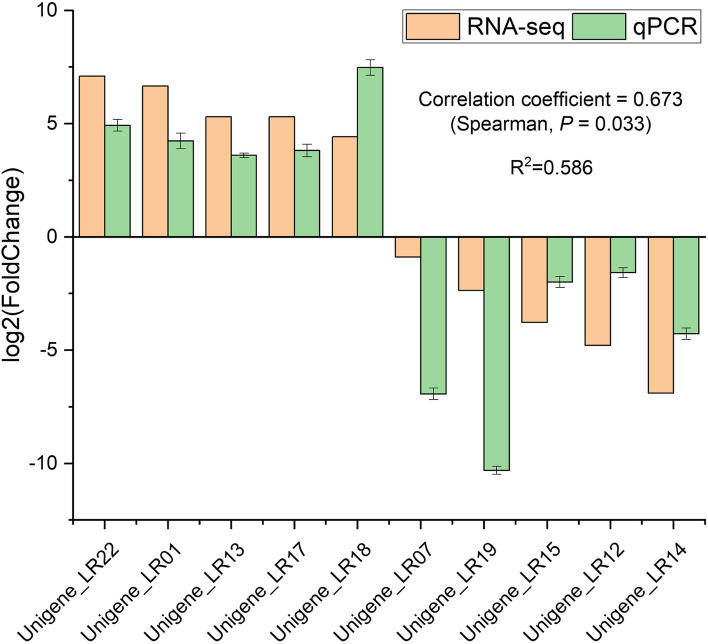
Validation of RNA-seq data using qPCR. The relative expression of genes from the RNA-seq analysis of MTP under salt stress was plotted against the RNA-seq (orange) and qPCR (green) data. R^2^ indicates the linear coefficient of determination between the RNA-seq and qPCR results

To validate the patterns of gene expression inferred from the RNA-seq experiments, 10 co-expressed genes in the leaves and roots were used for qPCR analysis (Fig. 9), including five up-regulated and five down-regulated genes. The expression patterns revealed by qPCR analysis of these genes (green bar) and RNA-seq data (orange bar) were similar. The positive Spearman correlation coefficient (*P* = 0.033, R^2^ = 0.586) between the qPCR and RNA-seq data was highly significant, indicating that our RNA-seq data were robust and could be used in subsequent analyses.

## Discussion

### MTP inherited high salt tolerance from *T. dactyloides* and *Z. perennis* and serves as a genetic bridge that will facilitate the genetic improvement of maize

Improving the stress resistance of crops to enhance crop yields via wild germplasm resources is essential under the background of future climate change. Soil salinization has greatly inhibited crop production, especially maize production, as maize is a glycophyte plant that is hypersensitive to salinity stress [47], and a 40% decrease in total plant biomass was observed when NaCl levels were between 100 mM and 150 mM NaCl. The salt tolerance of wheat [48] and rice [49] has been improved using wild relatives in previous studies, but such studies have been rarely conducted in maize. There is an urgent need to develop salt-tolerant maize germplasm. The maize wild relative teosinte and *T. dactyloides* are resistant to many types of abiotic and biotic stress [11]. Although *T. dactyloides* has the potential to be used to improve the salt tolerance of maize [20, 21], previous experiments have been conducted in vitro, and fertile offspring have not been successfully produced. In allopolyploids, traits can either be inherited from their ancestors via their parents or generated via polyploidization; consequently, a wide range of phenotypes are observed [50]. Polyploidization can promote species diversification; polyploids can also act as genetic bridges that permit the breakage of distant hybridization barriers and the introduction of foreign genes into crops from wild relatives [26, 27]. Iqbal et al. [30] previously reported a synthesized allopolyploid MTP that possesses the genomes of *Z. mays*, *T. dactyloides*, and *Z. perennis*; followed by the backcross lines BC_1_ and BC_2_, which were created by backcrossing with the maize inbred line. In this study, we evaluated the salt tolerance of MTP, and MTP exhibited higher salt tolerance than maize; its salt tolerance was even stronger than that of *T. dactyloides* and *Z. perennis* under 300 mM salt stress, which was indicated by its high growth rate (Fig. 1) and low Na^+^:K^+^ (Fig. 2). The high salt tolerance of MTP was thus inherited from *T. dactyloides* and *Z. perennis*; its salt tolerance even increased during the hybridization and polyploidization process, and this allows it to be used as a gene “pool” to improve the salt tolerance of maize. When used as a donor parent, high salt tolerance can also be observed in its offspring. We continued backcrossing the aforementioned backcross lines with maize inbred until there were no unfavorable traits (such as tillers), and then self-crossing was performed until plants were homozygous; this resulted in a series of MTP–maize introgression lines (BC_4_S_8_) with excellent characteristics. We identified many salt-tolerant introgression lines; Fig. S13 shows one of the salt-tolerant introgression lines, and salt-related gene mapping was carried out (unpublished data). Thus, MTP is a genetic bridge that permits the breakage of the hybridization barrier between modern maize and its polyploid wild relatives and thus the introduction of beneficial traits from *T. dactyloides* and *Z. perennis*.

### SMRT sequencing of MTP provides abundant sequence resources for the genetic improvement of maize

The lack of high-quality genomes has greatly limited the use of wild species for the enhancement of crops. Teosinte and *T. dactyloides* provide important secondary and tertiary gene pools [51]. The potential for sequences from *Z. perennis* and *T. dactyloides* to aid both basic biological research and be applied in maize breeding has long been discussed [22, 52]. However, due to the complexity of their genomes and the fact that these two polyploid species have not been used frequently for the genetic improvement of maize, there are only 1041 and 132 nucleotide sequences in the NCBI database for *T. dactyloides* and *Z. perennis* to date, respectively. The newly synthesized allohexaploid MTP, which has undergone hybridization and polyploidization and integrates the genomes of the three species, provides an effective genetic bridge between *T. dactyloides*/*Z. perennis* and maize [30]. Comprehensive genetic and genomic information on MTP will aid the identification and utilization of favorable alleles derived from *T. dactyloides*/*Z. perennis* or novel alleles that could be used in modern maize breeding. SMRT sequencing provides an unprecedented opportunity to identify full-length transcripts, especially for species without a reference genome [2, 34, 53]. In this study, we identified 227,375 full-length reference transcripts with an average length of 2300 bp across multiple tissues to provide an accurate and comprehensive transcriptome of MTP via SMRT sequencing.

We found that the mapping ratio (83.76%) of MTP against maize was lower than that of *T. dactyloides* (98.04%). This possibly stems from the large number of new sequences caused by hybridization and polyploidization and is one reason why a large number of sequences cannot be annotated. In addition, we identified 12,982 lncRNAs, and only 1826 lncRNAs showed high sequence similarity with maize; this provides more extensive lncRNA resources for the genetic improvement of maize. Because of the lack of genome information on *T. dactyloides* and *Z. perennis*, we cannot distinguish the ancestral type of our unigenes; this seriously limits the ability to identify the homeolog expression bias of MTP under salt stress. Additional research is needed to recognize homologous genes and characterize their expression bias. Phylogenetic analysis showed that MTP was more closely related to *T. dactyloides* and *Z. mexicana* than to maize (Fig. 4), which indicates that MTP possesses the genomes of three species and that the genomes of *T. dactyloides* and *Z. perennis* might have a greater influence on the phenotypic features of MTP than on the phylogenetic features of *Z. mays*. Genes with a high Ka/Ks ratio (> 1) were involved in “Ion channels,” “Environmental adaptation,” and “Protein families: signaling and cellular processing.” which are all related to plant stress resistance. Genes involved in “Ion channels” mediate the response to salt stress. These results suggest that these genes have likely experienced strong positive selection following hybridization and polyploidization and are involved in responses to environmental stimuli and stress. These data provide substantial sequence resources for the genetic improvement of maize.

### Molecular mechanism of salt tolerance regulation in MTP

Elucidating the mechanism of salt tolerance in maize and the identification of genes involved in salt tolerance have long been major goals of maize research. In this study, we performed an RNA-seq analysis of MTP under different salt concentrations using the full-length transcripts as a reference. The number of DEGs rapidly increased with the salt concentration, and more DEGs were observed between leaves and roots (Fig. S6). We identified 484 and 1035 DEGs that were co-induced under different salt concentrations in the leaves and roots, respectively, and the number of DEGs expressed increased with the salt concentration. This suggests that the expression of genes in MTP was rapidly induced by high salt stress. The number of genes that responded to salt stress was greater in the roots than in the leaves. A similar pattern has also been observed in a previous study of salt stress in a diploid wild relative of sweet potato [54].

Polyketide synthases play an important role in the biosynthesis of various plant secondary metabolites and the adaptation of plants to stress. Flavonoids are important secondary metabolites that mitigate the cytotoxic effects of ROS through the scavenging of free radicals [55]. Previous studies have shown that the accumulation of flavonoids can balance Na^+^/K^+^ ions to enhance plant salt tolerance [56]. In our study, the up-regulated genes in the roots were significantly enriched in the “Polyketide synthases,” “Flavonoid biosynthesis,” and “Unsaturated fatty acids biosynthesis” pathways (Fig. 6D). “Oxidoreductase activity,” “Glutathione gamma-glutamyl cysteinyl transferase activity,” and “Phosphatidylinositol 3-kinase binding” in leaves and “Oxidoreductase activity” in roots were also identified by DAG analysis (Figs. S7 and S9). This indicates that redox regulation plays a key role in both the leaves and roots. A recent study showed that the overexpression of serine carboxypeptidase in *Arabidopsis thaliana* significantly enhances salt tolerance, and the accumulation of proline induced by serine carboxypeptidase directly contributes to its salt tolerance [57, 58]. DEGs in the roots are also involved in serine-type carboxypeptidase activity. Our results indicate that serine-type carboxypeptidase plays an important role in the response of MTP to salt stress. Salt stress induces the expression of many stress-responsive genes, and the expression of these genes shows high spatio-temporal specificity. In the leaves, the expression of nine genes was up-regulated under low salt concentrations but down-regulated under high salt concentrations (Fig. 5H), and these genes were enriched in “Photosynthesis antenna proteins” and “Porphyrin and chlorophyll metabolism” pathway (Fig. S8). This result indicates that the enhancement of photosynthesis may protect MTP from the deleterious effects of low salt stress. However, photosynthesis was inhibited as salinity levels increased.

In the roots, the expression of 34 genes was up-regulated under low salt concentrations but down-regulated under high salt concentrations, including four TF families, the WRKY, B3-ARF, bHLH, and LOB TF families, and the cytokinin signal regulator ARR-A (Fig. 6H). These genes were all enriched in the “Plant hormone signal transduction” pathway (Fig. S10). Plant hormones play vital roles in plant growth, development, and the response to environmental stress [59]. Plants perceive stress signals, which trigger signal transduction cascades and downstream defense responses. Plant hormones mainly act through signal transduction pathways [60]. TFs mediate the perception of salt stress and regulate the expression of target genes by binding to the cognate *cis*-elements in the promoters of stress-related genes [61]. In our study, the TFs or response regulators above were involved in the hormone receptors of ABA, IAA, CTK, and JH [62–65], respectively. This indicates that the roots of MTP may first perceive salt stress signals and induce the expression of TFs, which induces the release of plant hormone signals to trigger the activation of downstream defenses. The LOB domain (*LBD*) gene is a plant-specific TF that plays an important role in plant growth and development [66]. Recent studies have shown that LOB not only regulates plant organ development but also plays important roles in plant growth and development, such as hormone responses, plant defense, leaf development, and root development [67–69]. In our study, the expression of a LOB TF was down-regulated under low salt stress but up-regulated under high salt stress. We speculated that genes encoding LOBs play key roles in regulating the salt tolerance of MTP.

To clarify the responses of various genes to changes in salinity in the leaves and roots, we carried out an expression pattern analysis using STEM software. A total of four similar significant expression patterns (*P* < 0.05) were identified in the leaves and roots under different salinity levels (Fig. 7A, B). The “Spliceosome” pathway was detected in all four expression patterns in both leaves and roots (Fig. 7C, D). Pre-mRNA splicing mediated by the spliceosome is an essential step between transcription and translation; the precision and efficiency of pre-mRNA splicing directly affect gene function. *SKIP* and *U1A* confer osmotic and salt tolerance by controlling the AS of genes in *Arabidopsis* [70, 71]. Therefore, we speculate that post-transcriptional regulation is particularly important in mediating the response of MTP to salt stress. DEGs in pattern 1 were specifically enriched in the “MAPK signaling pathway” and “Starch and sucrose metabolism,” and DEGs in pattern 3 were specifically enriched in “Carbon metabolism” pathways (Fig. 7C, D). Thus, the MAPK signaling pathway is sensitive to salinity and activated under low-salinity stress; it thus plays an important role together with energy metabolism in regulating salt tolerance.

HSPs are a family of proteins produced by cells in response to stressful conditions that play an important role in responses to biotic and abiotic stress [72]. HSPs scavenge reactive oxygen species (ROS) and enhance membrane stability by positively regulating the antioxidant enzyme system. Previous studies revealed many HSPs involved in salt tolerance in wheat [73], rice [74], and soybean [75]. According to the results of the PPI analysis in our study, most of the genes located at the core position in the largest subnetwork (Fig. S12A) encoded HSPs (HSPA, HSP90, and HSPA4), and the expression of these genes was up-regulated. These results indicate that HSPs play an important role in the response of MTP to salt stress. Unigene-L26, which has the highest node degree, was annotated to the *T. dactyloides* genome sequence in the NT database and encodes the 40S ribosomal protein. A previous study suggested that a ribosomal protein (AgRPS3aE) confers salt tolerance in yeast, and overexpression of *AgRPS3aE* significantly alleviates stress symptoms in *Arabidopsis* and tobacco [76]. This indicates that Unigene-L26 may have been inherited from *T. dactyloides* and that it regulates salt tolerance by interacting with other genes.

The expression of *RCI2A*, *ABF2*, and *ABF3* was co-expressed in the leaf, root, phloem, and xylem tissue of *P. euphratica* under salt stress, and these genes may play important roles in plants under salt stress [77]. In our study, 21,431 and 28,517 unique DEGs in the leaves and roots were identified, respectively (Fig. 8A), suggesting that the tissue-specific expression of genes in the roots and leaves was particularly pronounced and that these genes play distinct roles in the response to salt stress. A total of 22 DEGs expressed under different salt concentrations were detected in both the roots and leaves, but their expression patterns in the leaves and roots were not completely consistent across all salt concentrations (Fig. 8B, C). This indicates that these genes were conserved and that they might play complementary regulatory roles under stress. Such genes that function in multiple tissues simultaneously might be important for the breeding of salt-tolerant maize varieties.

## Conclusions

We showed that MTP has high salt tolerance and that it could be used as a genetic bridge to overcome the limitations associated with a lack of salt-tolerant germplasm resources in maize. By combining SMRT and RNA sequencing, we developed a comprehensive full-length reference transcriptome of MTP, which provides a novel, rich gene “pool” for maize. In addition, a series of DEGs involved in salt tolerance might provide genetic resources that could be used for the genetic improvement of maize. In sum, these results provide valuable genetic resources that will aid the utilization of maize wild relatives.

## Supplementary Information

**Additional file 1: Fig. S1.** Putative of transcription factors (TFs) and transcriptional regulators (TRs) by iTAK. **Fig. S2.** Prediction of long-non-coding RNAs (LncRNA) by CPC (Coding Potential Calculator), CNCI (Coding-Non-Coding Index) software and pfam protein structure domain database. **Fig. S3.** MTP Iso-Seq data mapped onto the maize reference genome (RefGen.v4). (A) 10 chromosomes of maize. (B) Transcript density of *T. dactyloides* in each chromosome. (C) Transcript density of Teosinte (D) Gene density of sorghum in each chromosome. (E) Transcript density of MTP in each chromosome. (F) Density of syntenic gene pairs between maize and *sorghum* in each chromosome. (G) Density of syntenic gene pairs between maize, *sorghum*, and MTP. (H) Distribution of lncRNAs in MTP. **Fig. S4.** Plot of expression level distribution of unigenes in each sample in FPKM intevals under gradient concentrations NaCl stress. (A) Gene expression level in leaf. (B) Gene expression level in root. Different shade of color represents different expression levels: FPKM < 0.3, 0.3–1, 1–50, > 50 represent a gene has a very low, low, high, very high expression level, respectively. **Fig. S5.** Cluster heat map showing the global relationships of the expressed genes in different samples and repeated experiments. **Fig. S6.** Count of DEGs numbers in each comparison group under salt stress. **Fig. S7.** Directed acyclic graph (DAG) for differentially expressed genes (DEGs) in the Molecular Function (MF) ontology in leaves. **Fig. S8.** KEGG pathway analysis of concentration specifically expressed genes in leaves. **Fig. S9.** Directed acyclic graph (DAG) for differentially expressed genes (DEGs) in the molecular function (MF) ontology in roots. **Fig. S10.** KEGG pathway of lower concentration specifically expressed genes in roots. **Fig. S11.** KEGG pathway analysis of higher concentration specifically expressed genes in roots. **Fig. S12.** Protein–protein interaction (PPI) network for DEGs in leaves (A)–(G) and roots (H)–(R). The different colors indicate the magnitude of log_2_ (foldchange). Circles and triangles represent interacting genes and transcription factors, respectively. **Fig. S13.** Comparison of salt tolerance between MTP-maize Introgression line 044 (IL044) and its recurrent parent inbred line (B73). Seeds were germinated and grown under distilled water (CK) and 200 mM NaCl stress (T) for 14 days.

**Additional file 2: Table S1.** SMRT sequences summary. **Table S2.** Statistics of unigenes unmapped to maize. **Table S3.** Functional annotation of unigenes unmapped to maize. **Table S4.** Orthologous groups in nine grass species according to maize (Mo17 and B73)-MTP-*T. dactyloides ortholog* relationships. **Table S5.** Statistics of orthologous gene set between MTP and nine grass species. **Table S6.** Summary statistics of RNA sequencing data. **Table S7.** Comparison of copy numbers of gene families in MTP that are involved in salt-saline tolerance with other species. **Table S8.** Expression patterns of genes in MTP that are involved in salt-saline tolerance.

## Data Availability

The datasets supporting the conclusion of this article are available in a publicly accessible repository (National Genomics Data Center). SMRT-seq and RNA-seq raw data can be found below: https://ngdc.cncb.ac.cn/gsa/s/7I6Qwv1A and https://ngdc.cncb.ac.cn/gsa/s/0m0jQ059. Other data are included within the article and its additional files.
