# Supplementary material for: Integrated single-molecule real-time sequencing and RNA sequencing reveal the molecular mechanisms of salt tolerance in a novel synthesized polyploid genetic bridge between maize and its wild relatives
Source: BMC Genomics. 2023 Jan 31;24:55. doi: 10.1186/s12864-023-09148-0 (PMC9887930; doi:10.1186/s12864-023-09148-0)
Supplement: Supplementary file 1 — Additional file 1: Fig. S1. Putative of transcription factors (TFs) and transcriptional regulators (TRs) by iTAK. Fig. S2. Prediction of long-non-coding RNAs (LncRNA) by CPC (Coding Potential Calculator), CNCI (Coding-Non-Coding Index) software and pfam protein structure domain database. Fig. S3. MTP Iso-Seq data mapped onto the maize reference genome (RefGen.v4). (A) 10 chromosomes of maize. (B) Transcript density of T. dactyloides in each chromosome. (C) Transcript density of Teosinte (D) Gene density of sorghum in each chromosome. (E) Transcript density of MTP in each chromosome. (F) Density of syntenic gene pairs between maize and sorghum in each chromosome. (G) Density of syntenic gene pairs between maize, sorghum, and MTP. (H) Distribution of lncRNAs in MTP. Fig. S4. Plot of expression level distribution of unigenes in each sample in FPKM intevals under gradient concentrations NaCl stress. (A) Gene expression level in leaf. (B) Gene expression level in root. Different shade of color represents different expression levels: FPKM < 0.3, 0.3–1, 1–50, > 50 represent a gene has a very low, low, high, very high expression level, respectively. Fig. S5. Cluster heat map showing the global relationships of the expressed genes in different samples and repeated experiments. Fig. S6. Count of DEGs numbers in each comparison group under salt stress. Fig. S7. Directed acyclic graph (DAG) for differentially expressed genes (DEGs) in the Molecular Function (MF) ontology in leaves. Fig. S8. KEGG pathway analysis of concentration specifically expressed genes in leaves. Fig. S9. Directed acyclic graph (DAG) for differentially expressed genes (DEGs) in the molecular function (MF) ontology in roots. Fig. S10. KEGG pathway of lower concentration specifically expressed genes in roots. Fig. S11. KEGG pathway analysis of higher concentration specifically expressed genes in roots. Fig. S12. Protein–protein interaction (PPI) network for DEGs in leaves (A)–(G) and roots (H)–(R). The diffe [file 12864_2023_9148_MOESM1_ESM.docx]

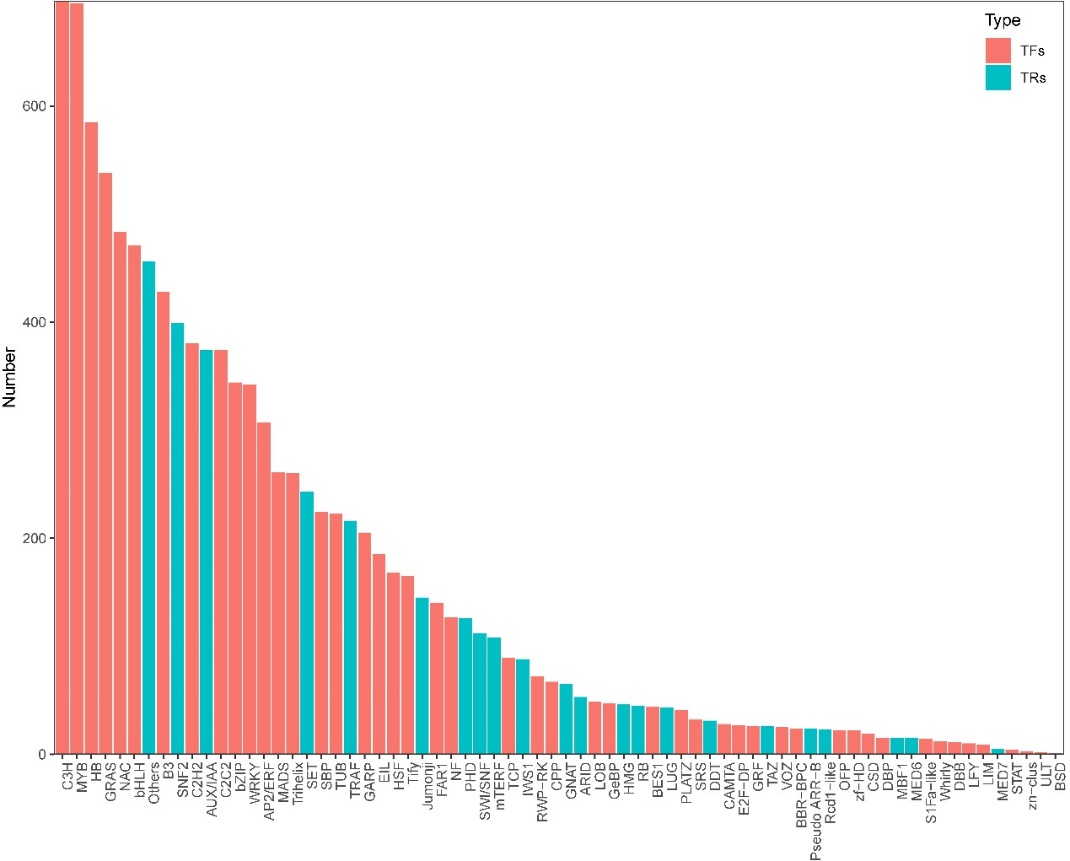


**Fig. S1**. Putative of transcription factors (TFs) and transcriptional regulators (TRs) by iTAK.


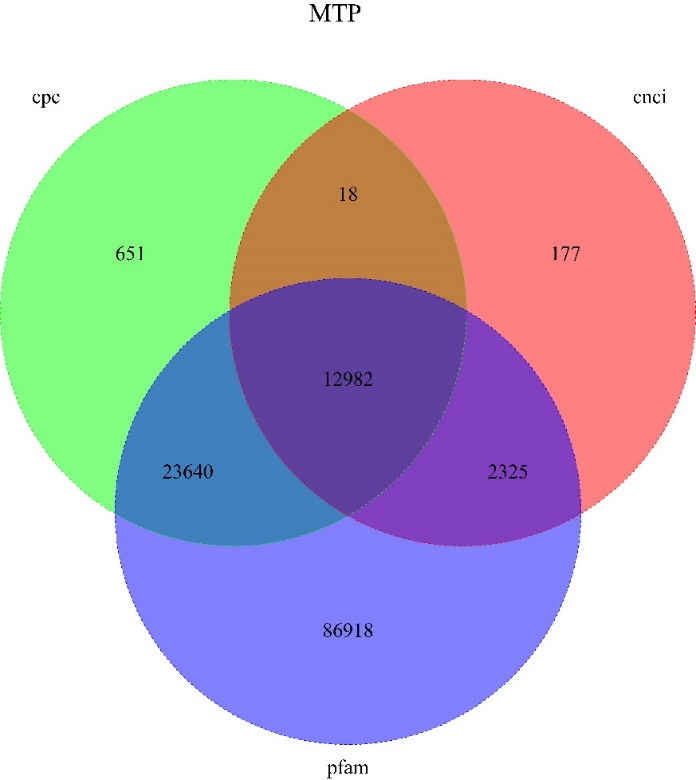


**Fig. S2**. Prediction of long-non-coding RNAs (LncRNA) by CPC (Coding Potential Calculator), CNCI (Coding-Non-Coding Index) software and pfam protein structure domain database.

**
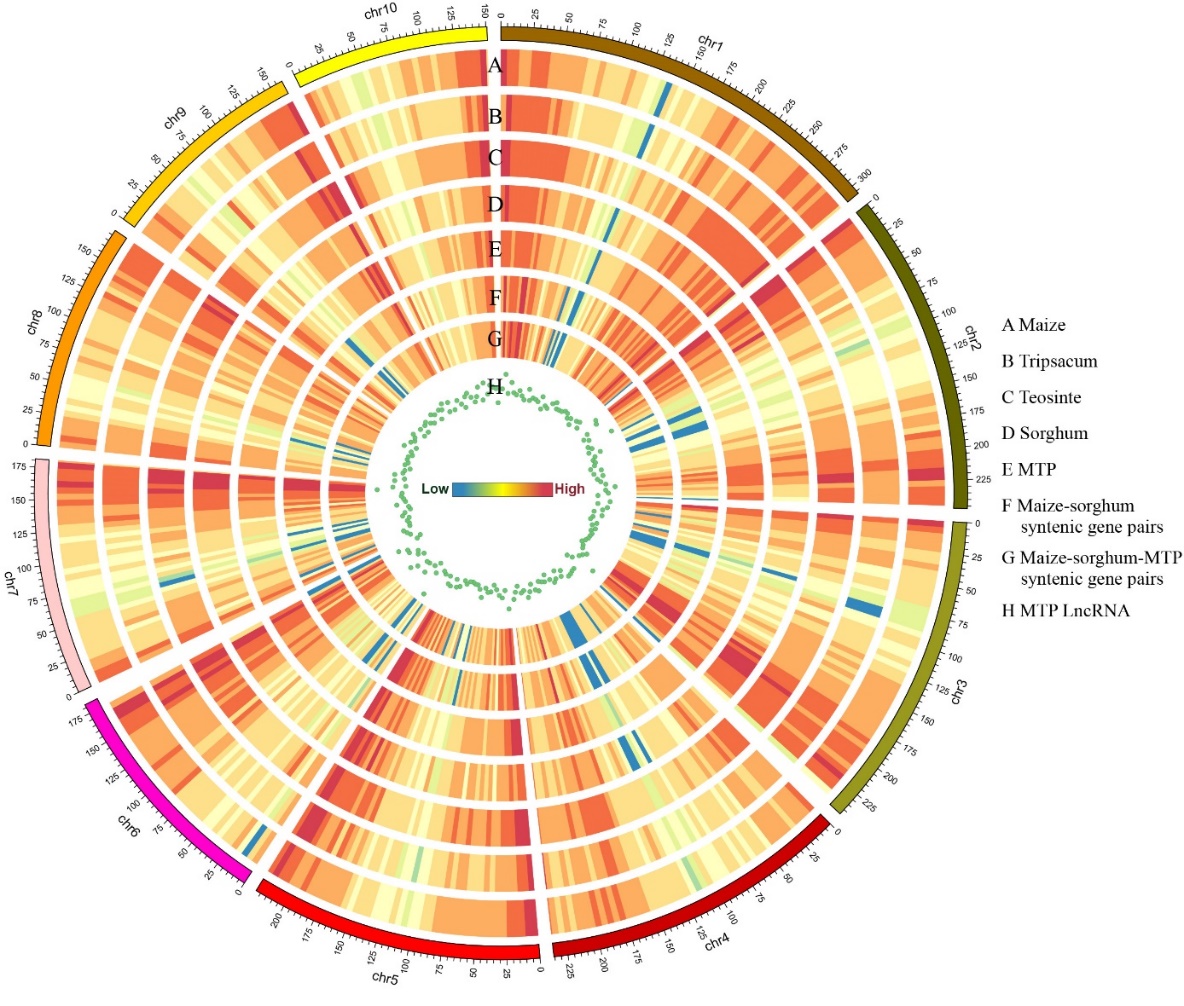
 Fig. S3.** MTP Iso-Seq data mapped onto the maize reference genome (RefGen.v4). **(A)** 10 chromosomes of maize. **(B)** Transcript density of *T. dactyloides* in each chromosome. (**C)** Transcript density of Teosinte **(D)** Gene density of sorghum in each chromosome. **(E)** Transcript density of MTP in each chromosome. **(F)** Density of syntenic gene pairs between maize and *sorghum* in each chromosome. **(G)** Density of syntenic gene pairs between maize, *sorghum*, and MTP. **(H**) Distribution of lncRNAs in MTP.


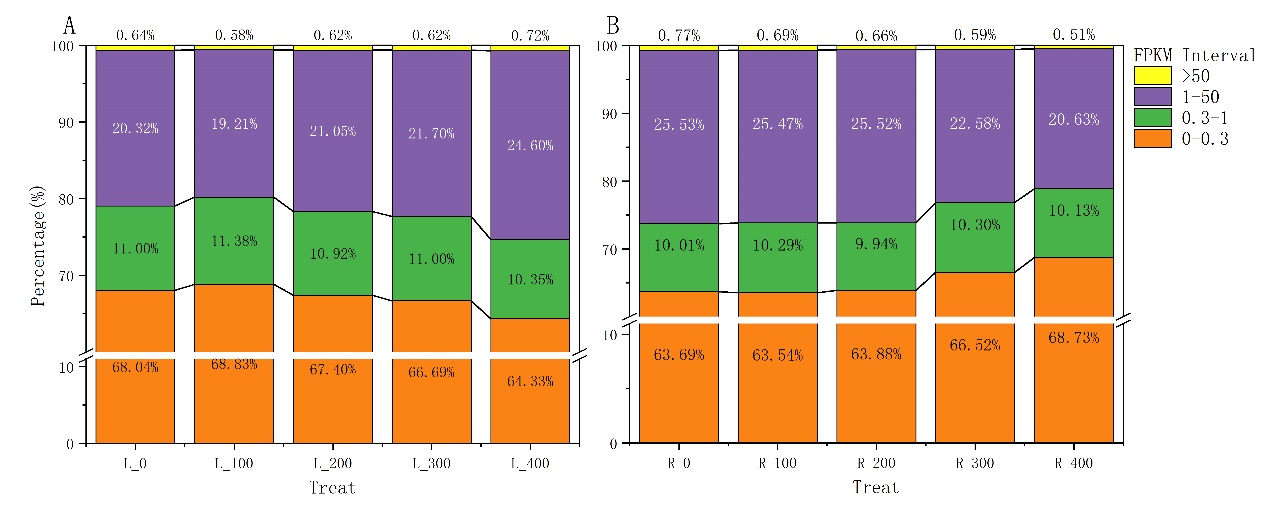


**Fig. S4**. Plot of expression level distribution of unigenes in each sample in FPKM intevals under gradient concentrations NaCl stress. **(A)** Gene expression level in leaf. **(B)** Gene expression level in root. Different shade of color represents different expression levels: FPKM < 0.3, 0.3-1, 1-50, >50 represent a gene has a very low, low, high, very high expression level, respectively.


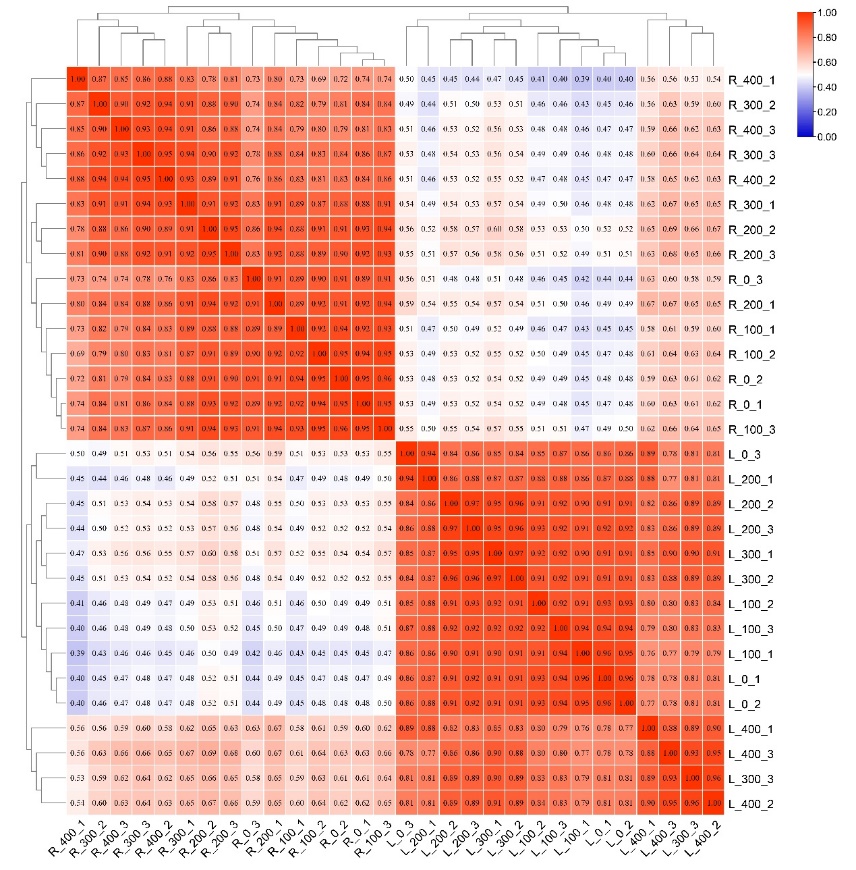


**
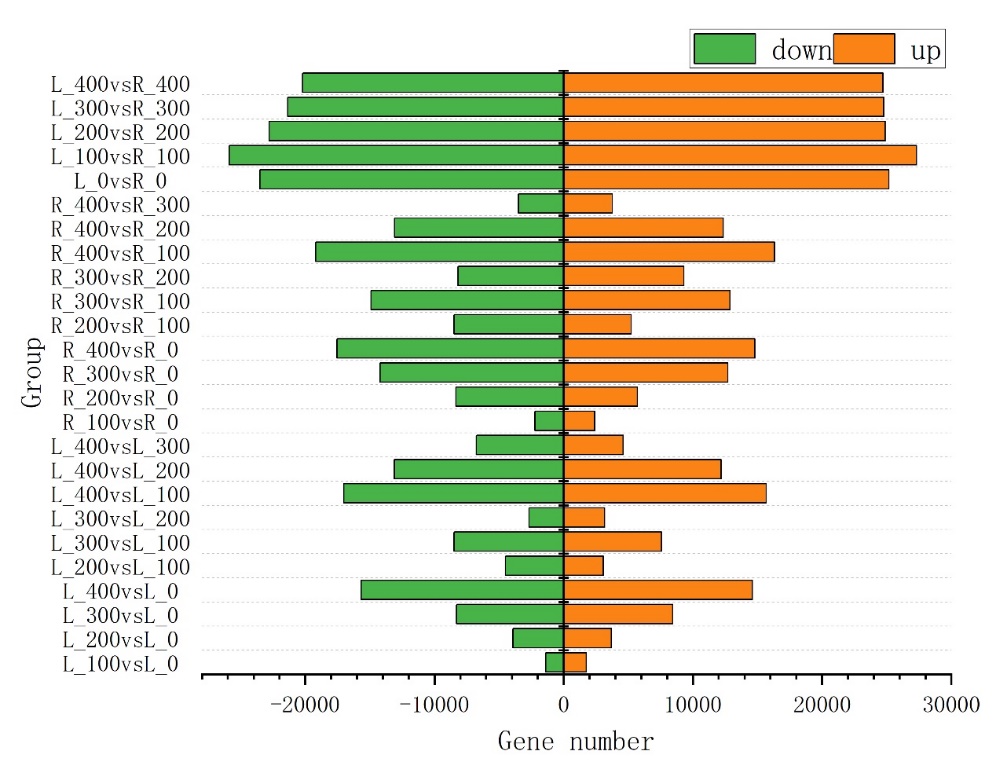
 Fig. S5.** Cluster heat map showing the global relationships of the expressed genes in different samples and repeated experiments.

**Fig. S6**. Count of DEGs numbers in each comparison group under salt stress.


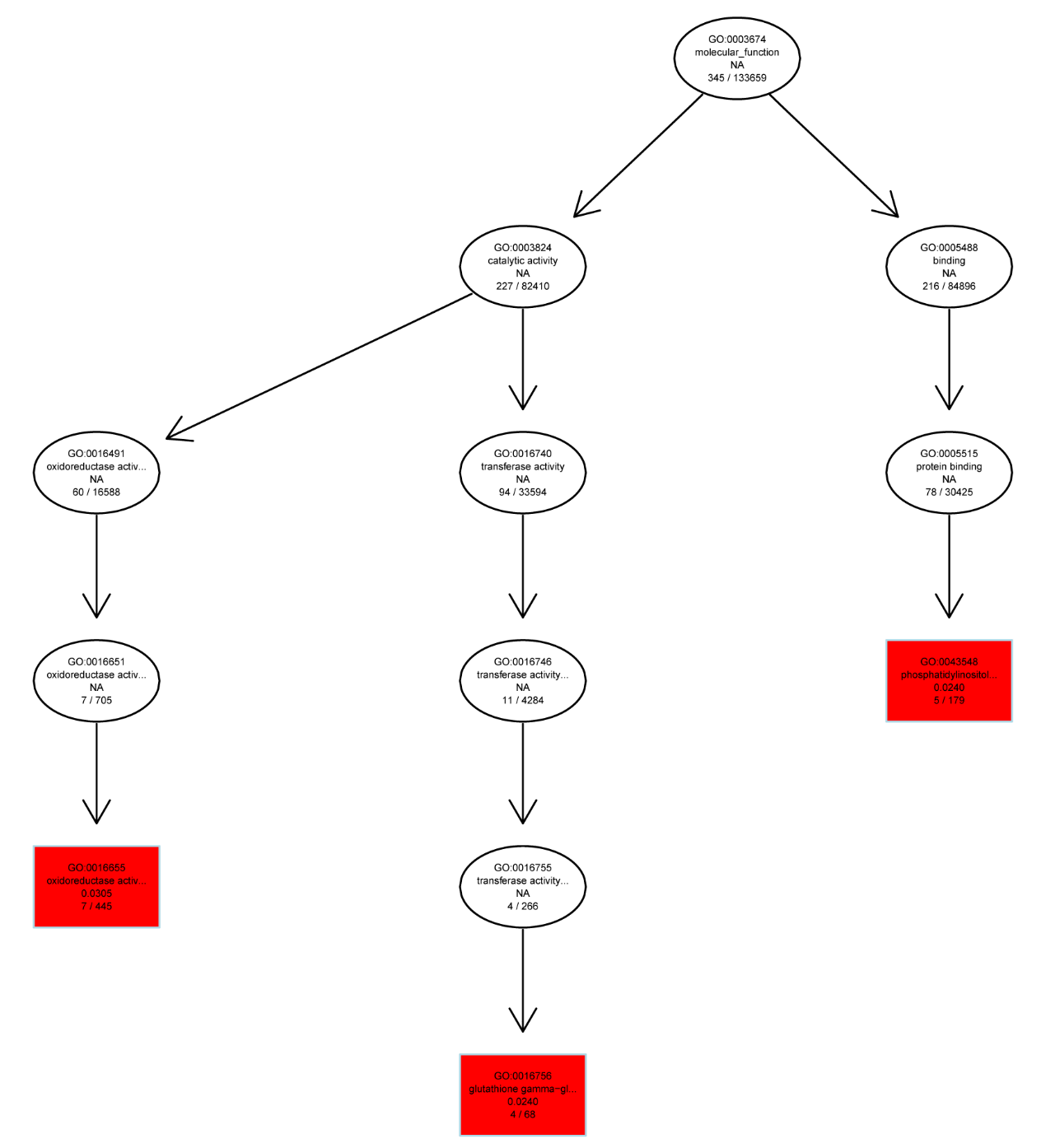


**Fig. S7**. Directed acyclic graph (DAG) for differentially expressed genes (DEGs) in the Molecular Function (MF) ontology in leaves.


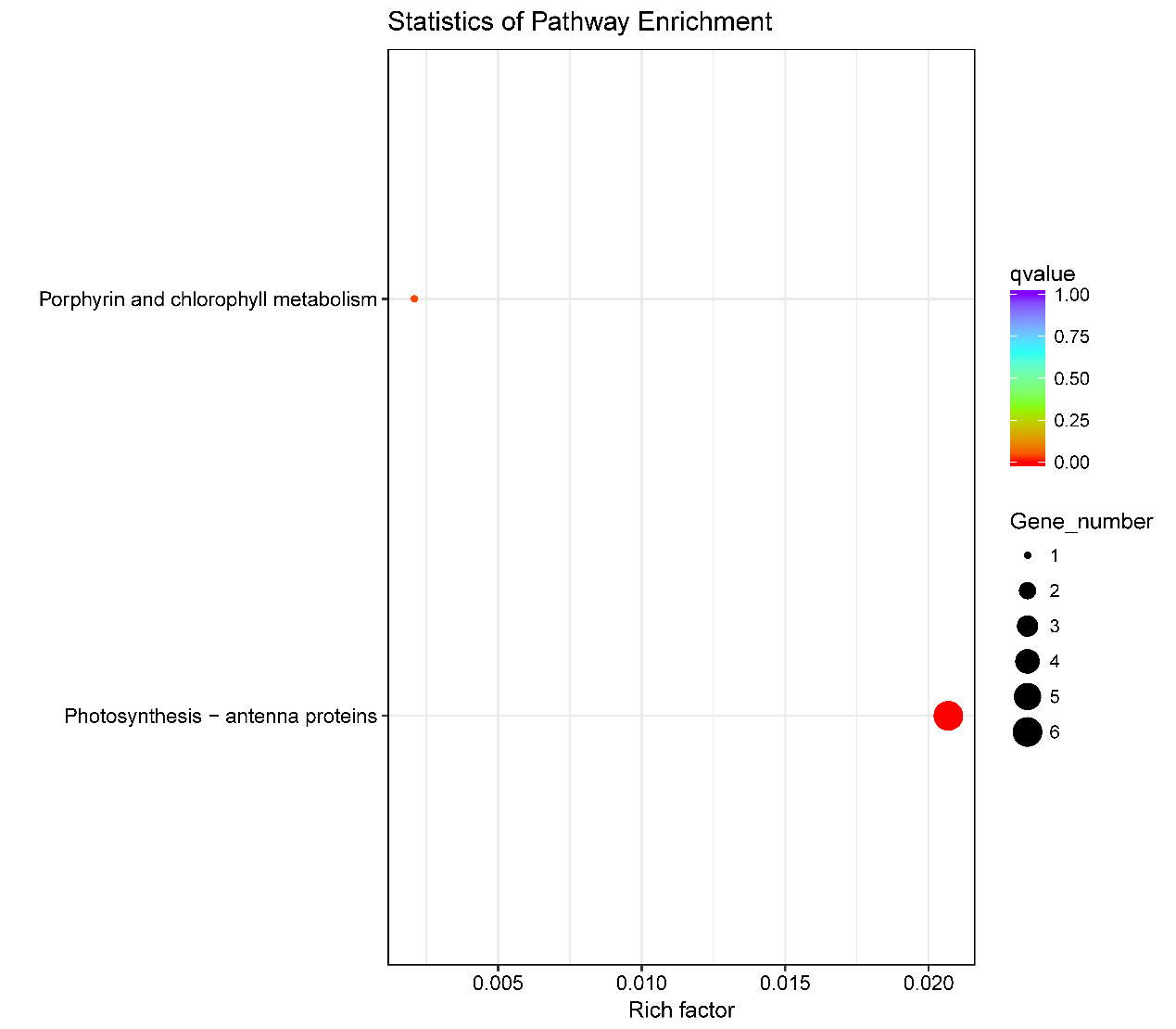


**Fig. S8**. KEGG pathway analysis of concentration specifically expressed genes in leaves


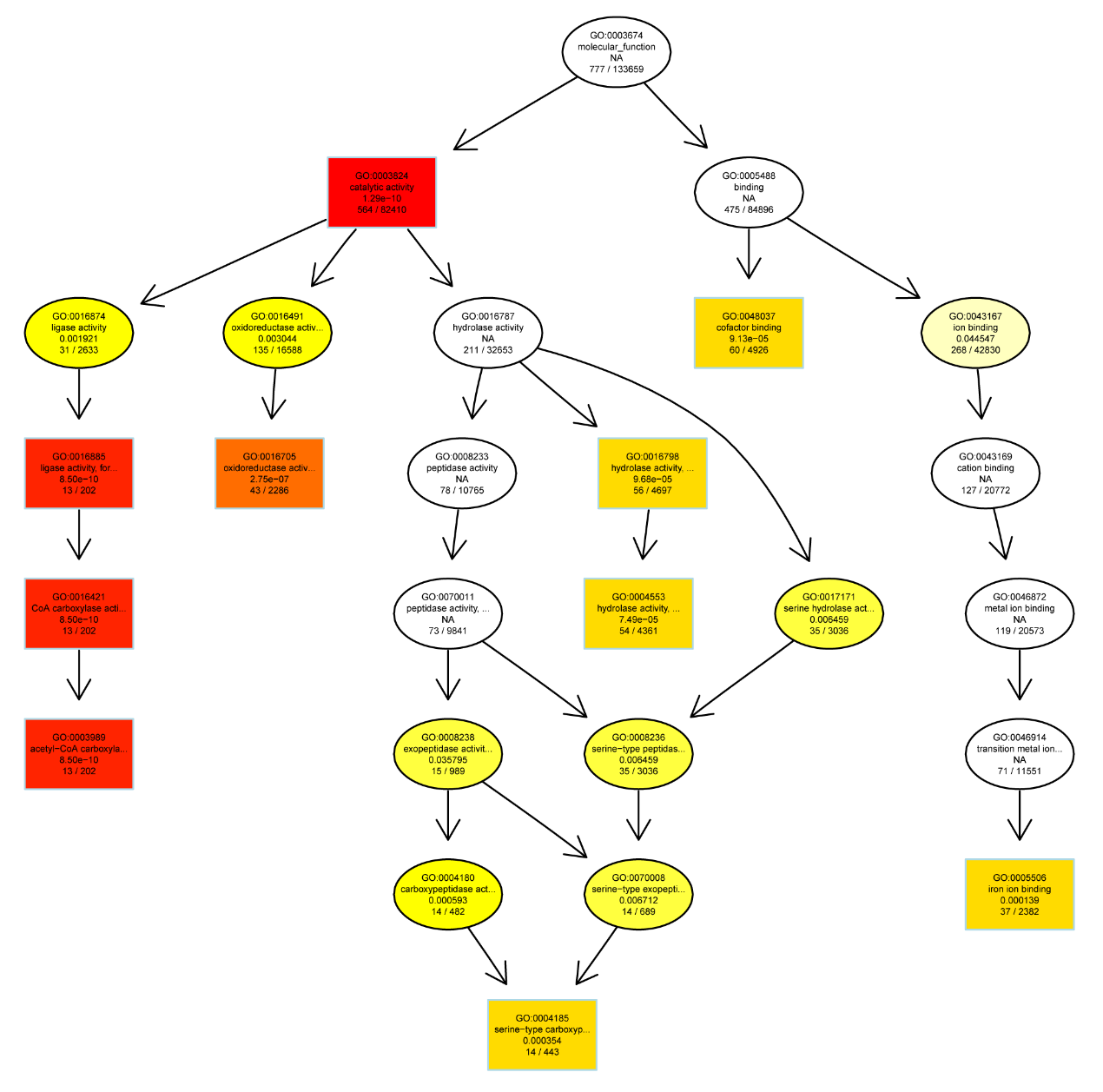


**Fig. S9**. Directed acyclic graph (DAG) for differentially expressed genes (DEGs) in the molecular function (MF) ontology in roots.


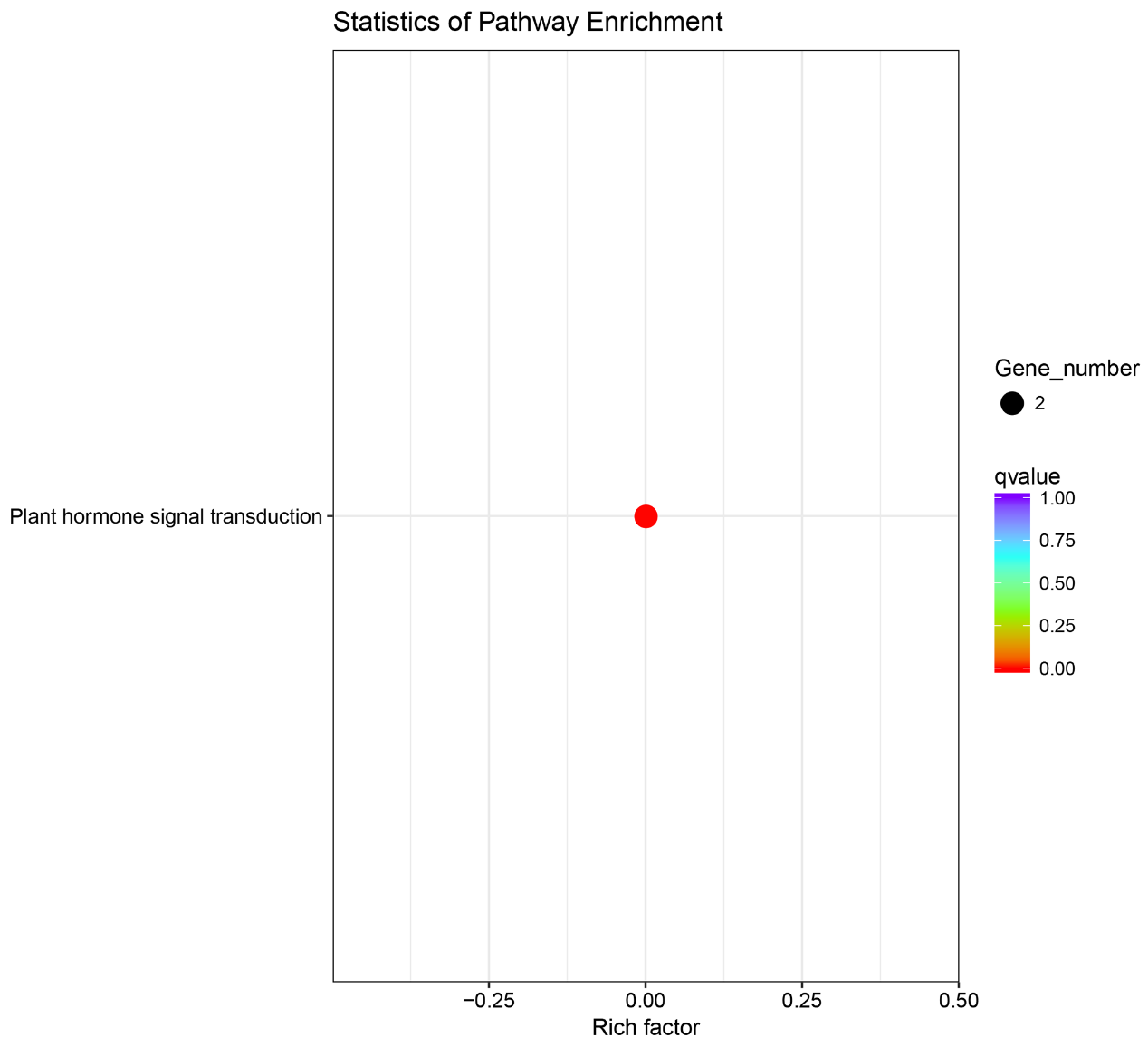


**Fig. S10**. KEGG pathway of lower concentration specifically expressed genes in roots


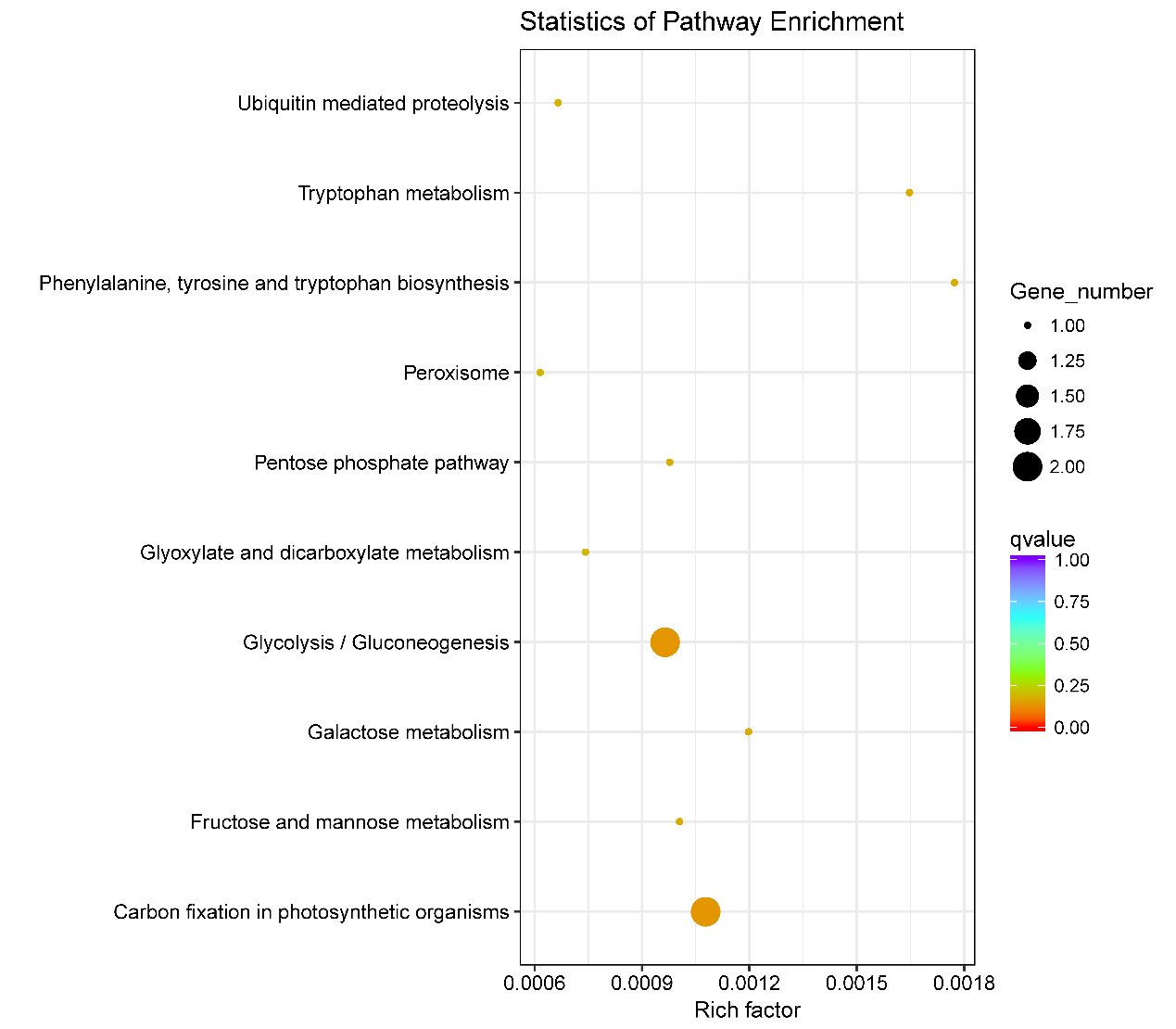


**Fig. S11**. KEGG pathway analysis of higher concentration specifically expressed genes in roots


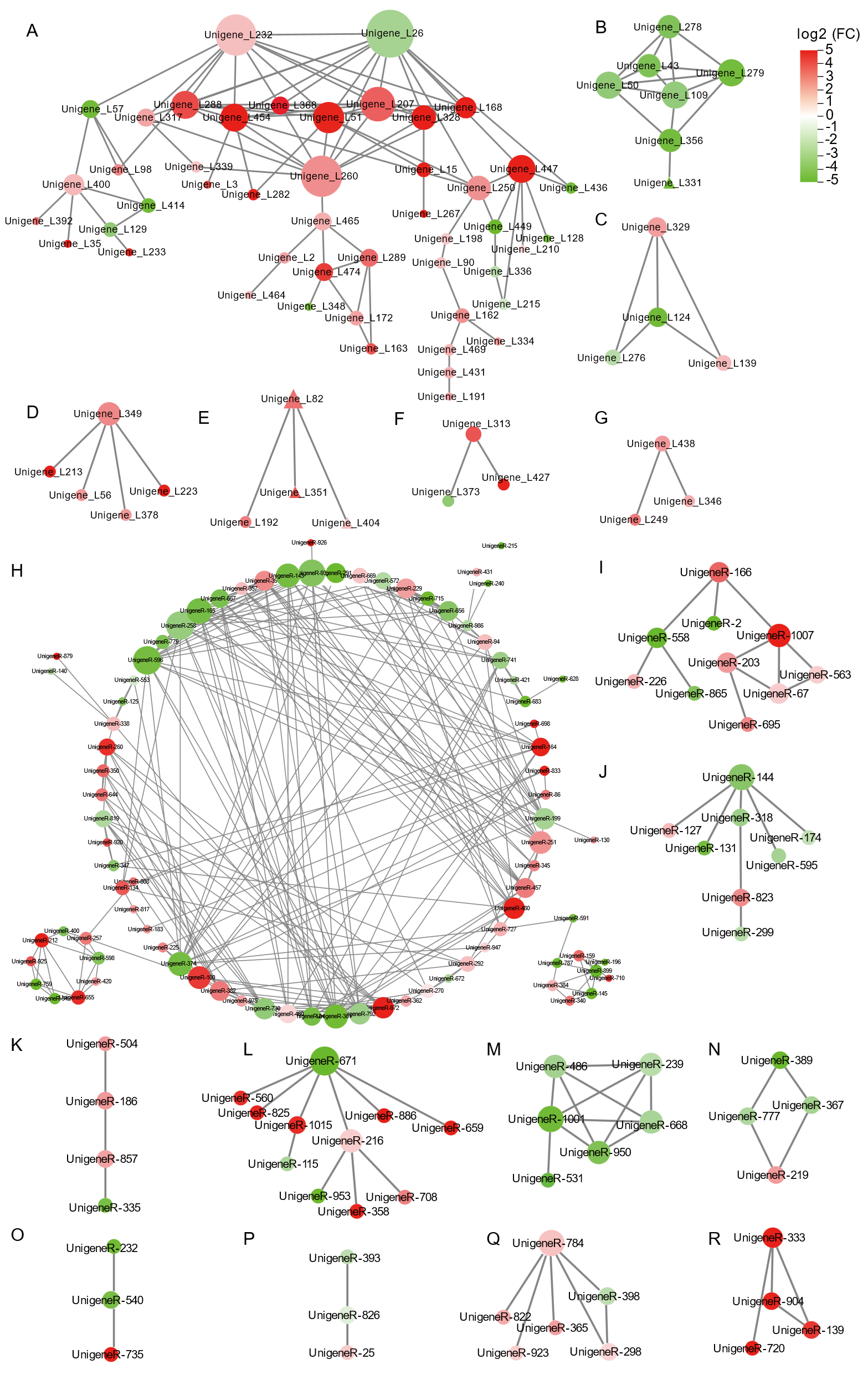


**Fig. S12**. Protein–protein interaction (PPI) network for DEGs in leaves **(A)**–**(G)** and roots **(H)**–**(R)**. The different colors indicate the magnitude of log_2_ (foldchange). Circles and triangles represent interacting genes and transcription factors, respectively.


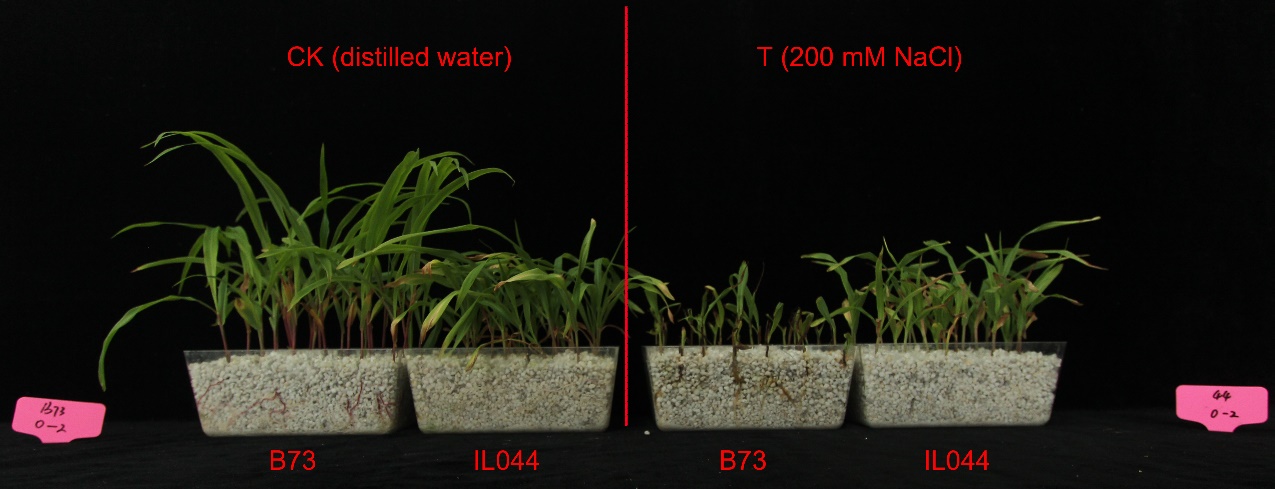


**Fig. S13**. Comparison of salt tolerance between MTP-maize Introgression line 044 (IL044) and its recurrent parent inbred line (B73). Seeds were germinated and grown under distilled water (CK) and 200 mM NaCl stress (T) for 14 days.
